# Three-Finger Toxins from Brazilian Coral Snakes: From Molecular Framework to Insights in Biological Function

**DOI:** 10.3390/toxins13050328

**Published:** 2021-04-30

**Authors:** Jessica Matos Kleiz-Ferreira, Nuria Cirauqui, Edson Araujo Trajano, Marcius da Silva Almeida, Russolina Benedeta Zingali

**Affiliations:** 1 Laboratório de Hemostase e Venenos—Instituto de Bioquímica Médica, Leopoldo de Meis (IBqM) and Instituto Nacional de Ciência e Tecnologia de Biologia Estrutural e Bioimagem (Inbeb)—Universidade Federal do Rio de Janeiro (UFRJ), Rio de Janeiro 21941-902, Brazil; jessica.kleiz@bioqmed.ufrj.br (J.M.K.-F.); edsontrajanoa@gmail.com (E.A.T.); 2 Protein Advanced Biochemistry (PAB), Instituto de Bioquímica Médica Leopoldo de Meis (IBqM) and Centro Nacional de Biologia Estrutural e Bioimagem (CENABIO), Universidade Federal do Rio de Janeiro, Rio de Janeiro 21941-902, Brazil; msalmeida@cenabio.ufrj.br; 3 Faculdade de Farmacia, Universidade Federal do Rio de Janeiro (UFRJ), Rio de Janeiro 21941-902, Brazil; cirauqui@pharma.ufrj.br

**Keywords:** *Micrurus* venoms, three-finger toxins, sequence variability, structure-function, classification guideline

## Abstract

Studies on 3FTxs around the world are showing the amazing diversity in these proteins both in structure and function. In Brazil, we have not realized the broad variety of their amino acid sequences and probable diversified structures and targets. In this context, this work aims to conduct an in silico systematic study on available 3FTxs found in *Micrurus* species from Brazil. We elaborated a specific guideline for this toxin family. First, we grouped them according to their structural homologue predicted by HHPred server and further curated manually. For each group, we selected one sequence and constructed a representative structural model. By looking at conserved features and comparing with the information available in the literature for this toxin family, we managed to point to potential biological functions. In parallel, the phylogenetic relationship was estimated for our database by maximum likelihood analyses and a phylogenetic tree was constructed including the homologous 3FTx previously characterized. Our results highlighted an astonishing diversity inside this family of toxins, showing some groups with expected functional similarities to known 3FTxs, and pointing out others with potential novel roles and perhaps structures. Moreover, this classification guideline may be useful to aid future studies on these abundant toxins.

## 1. Introduction

In Brazil, the *Micrurus* genera of coral snakes represents almost the totality of elapids, counting about 34 species [1] widespread throughout the country. Just as other coral snakes, the venoms of Brazilian *Micrurus* are predominant in three-finger toxins (3FTxs) and phospholipase A_2_ (PLA_2_), along with other less abundant proteins [2,3,4]. They may cause several injuries on envenomed animals such as myotoxicity, edema, nephrotoxicity, hemorrhages, and neurotoxicity. In humans, the major harm inflicted by these venoms is the blockage of neuromuscular junction by the action of pre and post-synaptic toxins, the PLA_2_s and the 3FTxs, which may lead to an outcome of respiratory arrest [5,6].

The 3FTxs belong to a family of non-enzymatic proteins constituted by approximately 58 to 90 amino acid residues. In all members of the family characterized to date, the protein fold is based on three loops of β-strands that resemble “fingers” extending to a globular core, stabilized by four conserved disulfide bridges [7]. These toxins present at least eight cysteines commonly well conserved, but also may present nine, ten, or even eleven cysteines [3,7]. This group of toxins is very exquisite and diversified in terms of primary structure and biological roles. This diversification has been proposed to be associated with the theory in which exonic regions in 3FTx genes have been exchanged by others. For instance, it can cause a shift in segments that may alter local structures and charge surface (accelerated segment switch in exons to alter targeting—ASSET mechanism) [8] generating the observed sequence diversity bounded to the multi-functionality and diversified targets. In concert, the prey–predator battlefield causes an evolutionary race, where negative and positive selection of key amino acids may also take place [9]. A wide array of biological roles is described for these toxins. One of the classical and well described activities is the agonism and antagonism of cholinergic receptors [10,11,12]. Updates in the last decade listed other 3FTx functions, including modulation of GABA A receptors [13,14,15], inhibition of acid-sensing ion channels (ASICs) [16], modulation of adrenoreceptors [17,18,19,20], activation of potassium channel [21], activation of voltage-gated sodium channel [22], activation of sperm-mobility [23], induction of insulin secretion from β-cells [24,25], among many others.

In the venoms from Brazilian *Micrurus* species, the amount of 3FTxs is very significant, even reaching 95% of the whole venom, as in the case of the *Micrurus surinamensis* species [26]. This represents a vast universe of possibilities of functions to be explored. Even though, few studies have been published to date about these 3FTxs from Brazilian *Micrurus* species. Considering the characterization of the 3FTxs in Brazilian venoms per se, meaning the identification of their molecular biology and functions, just a few articles were found, and these did not deepen the understanding of their function and molecular mechanism of action [27,28,29].

The last years of molecular biology and pharmacological studies on 3FTxs around the world in the frame of toxinology show all the diversity in amino acid sequence, structure, and function of these proteins. As mentioned, in Brazil barely anything is known, leaving a gap in the knowledge of these incredible toxins. In this way, this work aimed to conduct a systematic study on 3FTxs from Brazilian *Micrurus* species, focusing on their amino acid sequences and predicted 3D structures. By doing so, we bring insights on biological function, and also in the phylogenetic relationship of these toxins, which may guide further functional characterizations.

## 2. Results

### 2.1. Three-Finger Toxins Classification Based on Protein Threading

Three-dimensional protein structure undergoes evolutionary changes more ponderously than the protein sequence itself. This knowledge is well exemplified in the 3FTx family. They present a remarkably diversified primary structure, despite the overall three-dimensional conformation of “3 fingers” being conserved in all protein-members of this family. For this reason, we proposed a protein-classification based on a protein-threading algorithm, which takes into account conserved structural patterns (see methods).

We used the HHPred software to select the most probable structural homologue for each protein sequence used in the present study, by using an implemented algorithm for hidden Markov models comparison (HMM-HMM) in HHPred. This method allows sensitive detection of a structural homologue since it is based on profile-sequence comparison, not just sequence-sequence comparison [30,31,32,33].

As a first step, we grouped 57 sequences of our dataset (Table S1) according to the best hit with the lowest E-value suggested by HHPred, curated manually. To further verify and refine this first classification, and to get the final structural homologue, we analyzed conserved sequence-patterns, finger extension, and disulfide bridges in those sequences (Table S2). For example, we observed that some sequences have a prolongation of the middle finger, as in the case of group 8. Other examples are groups 2 and 7, in which we found a 5th extra disulfide bond in the first finger. Thus, we verified if sequences with distinguished structure were gathered into groups in line with HHPred results. Afterward, some groups were merged together as subgroups, due to the proximity both in homologous function-structure characteristics and in phylogenetic analysis. Moreover, the sequence length did not seem to be related to group separation according to this method-analysis, as we found a combination of sequences with different lengths grouped together.

**Figure 1:**
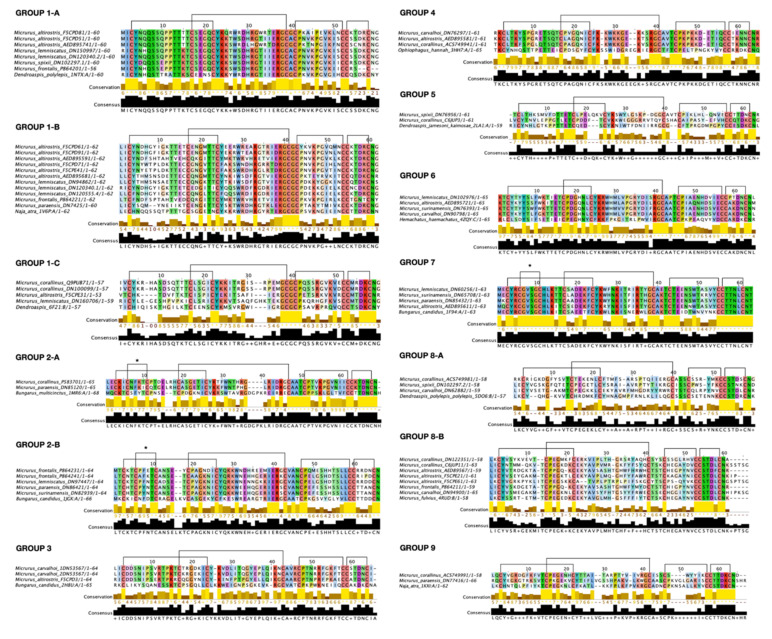
Sequence alignment of 3FTx groups. Groups are presented with their consensus amino acids and conservation score on a scale from 0 to 10 (10 being indicated by an asterisk as completely conserved, and + for similar amino acids). The cysteine residues forming the disulfide bridges are linked by black lines on the top of the alignment. The extra disulfide bridges are marked with an asterisk. The figures were prepared with the Jalview software [34] and colored using the Clustalx scheme and conservation.

**Figure 2:**
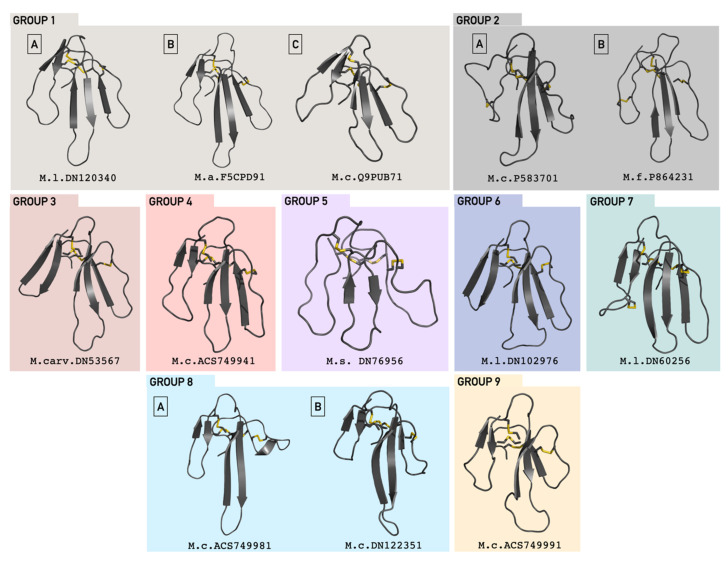
Representative 3D structural models of each 3FTx group. Each group and subgroup are represented by a 3D structure modeled from a representative sequence and identified by its database accession code. The best model was chosen according to the lowest DOPE score (*Group 1-A* -3469.3; *Group 1-B* -4223.5; *Group 1-C* -3663.6; *Group 2-A* -3945.3; *Group 2-B* -3770.5; *Group 3* -4401.7; *Group 4* -3720.2; *Group 5* -3608.5; *Group 6* -5266.9; *Group 7* -5035.04; *Group 8-A* -3868.4; *Group 8-B* -4417.3; *Group 9* -4235.3). The cysteine residues that form disulfide bridges are marked yellow. The structures are presented from the N-terminus to the C-terminus, and the fingers I, II and III are positioned from the left to the right. Colored boxes represent each group of toxins.

We ended up with a total of 9 groups, group 1 being subdivided into three subgroups (named 1-A to 1-C), group 2 subdivided into two subgroups (named 2-A and 2-B), and group 8 also subdivided into two subgroups (8-A and 8-B) (Figure 1, Figure 2 and Table S2).

### 2.2. Structural Representative Model

The knowledge of a protein structure is fundamental to understand its function. Taking this into consideration, we created a 3D model of one sequence from each group with the aim of identifying functional-structural signatures. Since the cysteine residues are extremely conserved in this family, we checked manually their pairing to verify the alignment trustworthiness and quality. A total of 13 representative models were created (Figure 2). Additionally, we performed an independent prediction of secondary structure in the JPred server, using solely the protein sequence, and comparing it with the secondary structures observed in our structural models. For almost all proteins, JPred estimated a β-strand conformation for fingers II and III (Figure S1). Curiously, JPred did not predict a β-strand for the finger I for most of the proteins. This finger could be more flexible than the others and it may experience transitory conformations.

We found an interesting exception for group 7 and subgroup 8-A, in which JPred predicted an α-helix in the finger II (significance score of 6 to 8), while the 3D models one β-sheet (Figure 2 and Figure S2). Until the present date, no α-helix has been reported in 3FTxs. More studies must be conducted to understand the conformational characteristics of finger II in these proteins.

The 3D models were also useful for the calculation of charge distribution on the protein surface, which is helpful to find conserved motifs for interaction with other molecular targets, for example. The surface charge distribution was calculated for each representative structure of each group (Figure S2).

### 2.3. Phylogenetic Analysis

**Figure 3:**
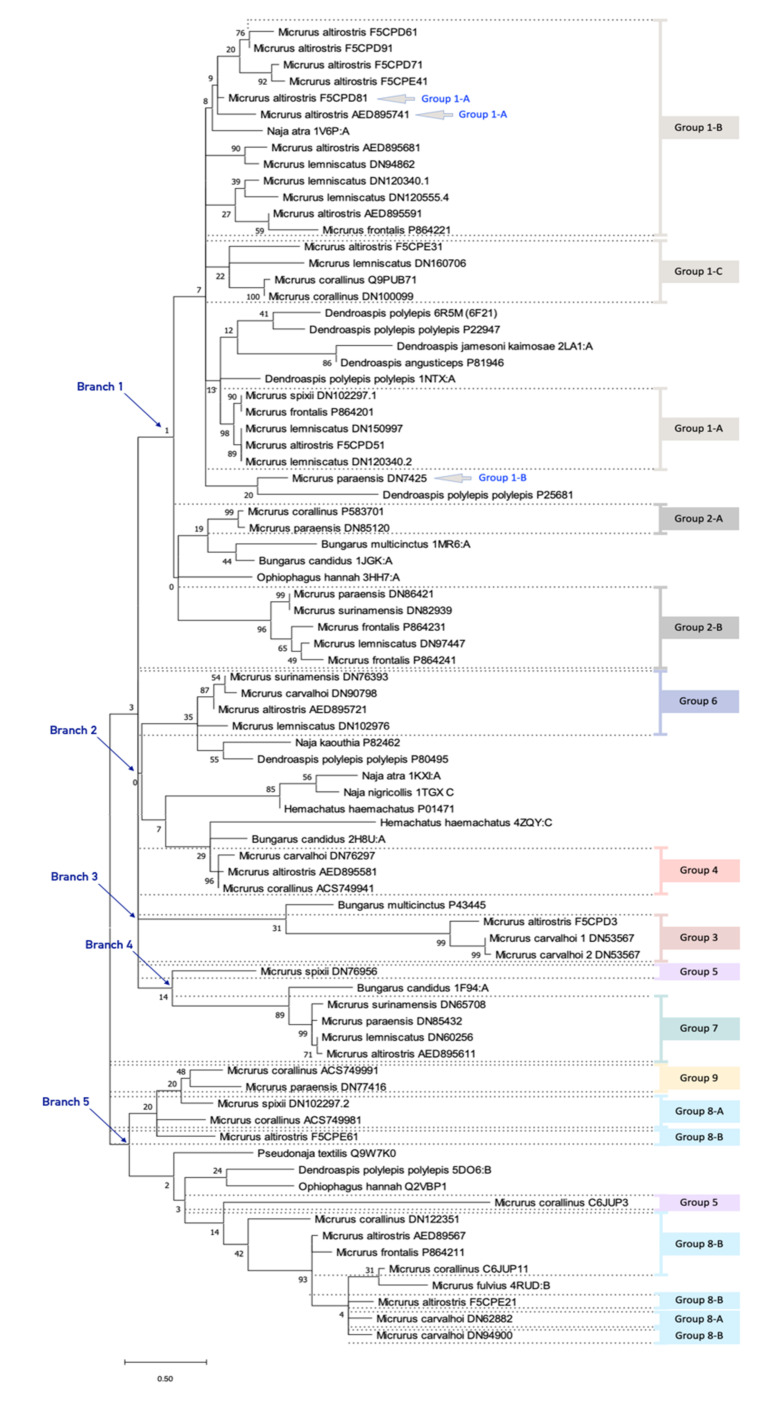
Phylogenetic tree of the 3FTxs sequence groups. The evolutionary analysis was generated using the maximum likelihood method and the Whelan and Goldman model. The tree with the highest log likelihood (−2630.25) is shown. The 3FTxs groups are indicated with colored boxes. 3FTxs already characterized were included in this analysis. The sequences found in the tree inside a different group from the one suggested in Figure 1 are indicated in blue text, with a grey arrow. The five big branches are shown here in dark blue.

Nowadays, the phylogenetic analysis is a useful tool not just to comprehend the relationships among species but also to make important inferences in almost every arm of biology, including molecular biology. In the present study, besides the structural analysis, we used the phylogenetic information to organize the groups and to compare with the classification based on the structural data (Figure 3). As this analysis is based on nucleic acids or amino acids alignment, and given the sequence variation in the 3FTx family, we have been expecting low amino acid matches in the alignment positions, afterward impacting the *bootstrap* of the phylogenetic tree. Indeed, we observed a very low *bootstrap* in the outermost tree nodes, almost all in the root of the big branches (branch 1 to branch 5). Also, the inclusion of some known old world 3FTxs as references impacted the *bootstrap* value. Interestingly, we found corroboration between the grouping by structural analysis and by the phylogenetic analysis. For instance, all sequences of group 1 are gathered together in the same branch. Moreover, we observed a similar classification into subgroups, with just three exceptions (marked by grey arrows and blue text in Figure 3).

### 2.4. Biological Insights Annotation

3FTxs possess vast modes of action, several molecular targets, and many functions. Alongside the conserved global 3D structure as already mentioned, these toxins evolved to keep a range of sequence variation inside their small and tight shape, to guarantee their biological versatility. To have insights about the molecular properties and to raise a hypothesis about the biological roles of 3FTxs from Brazilian *Micrurus* venoms, we started looking at the information of their structural homologous. We compared conserved regions in those sequences such as amino acids directly or indirectly involved in interaction with a functional partner, disulfide bridge patterns, local 3D conformation, and distribution of charges on the protein surface. Additionally, we made a data collection of 3FTxs already described in the literature and compared with our groups (Table S3).

## 3. Discussion

### 3.1. Brazilian Micrurus Potent Weapons—Molecular and Biological Insights

Since the publication of the transcriptomes of *Micrurus altirostris* and *Micrurus corallinus* species [2,35] and the recent production of venom gland transcriptomes of six Brazilian *Micrurus* taxa [3], the databank of toxin sequences from Brazilian *Micrurus* species was hugely upgraded. The availability of these toxins represents a great step to have an idea of the vast diversity inside the elapid venoms in Brazil. Despite the latest contributions, there is a lack of information about structure-function, which prompted us to utilize the current available sequences to start deepening the investigation of the molecular biology behind the *Micrurus* toxins by bioinformatic analysis.

We decided to explore one of the two most abundant and relevant toxins in *Micrurus* venoms, the three-finger toxins (3FTxs). As well as other 3FTxs in general, the ones from *Micrurus* venoms from Brazilian species have high diversity in terms of primary structure, conserving mainly the eight structural cysteines that anchor their globular core and the projection of three fingers composed usually by β-strands and loops (Figure S3). In order to separate and systematically study these proteins, we developed a guideline considering the high variability among their amino acid sequences. To establish our method, we assembled our database with selected sequences and sorted out the proteins initially based on structural homology identity. Then, we looked at other important patterns such as key functional amino acids in the primary structure, disulfide bridges, charge distribution on protein surface, and sequence phylogenetic relationship. Gathering these analyses, we manage to classify these toxins into groups (Figure 1).

Each proposed group will be discussed hereafter, pointing out the most important details that we extracted from all analyses, which sheds light on the molecular properties and possible biological roles of some 3FTxs from Brazilian *Micrurus* species. This guideline can also be useful for other classes of toxins that have diversified primary structures.

#### 3.1.1. Subgroup 1-A

One of the most important and well-conserved residues in α-neurotoxins for the activity in nicotinic acetylcholine receptor (nAChR) is the Arg 33, located in the outermost section of the finger II, (usually at position 33) [12,36], which is reported in the literature as being a site for invariant functional amino acids [37]. Noteworthy, other residues have been also pointed out in α-neurotoxins as important for activity, such as Lys 23/27, Trp 27/29, Asp 27/31, and Lys 47/49. For example, in the α-neurotoxin III from *Laticauda semifasciata* venom (LsIII), the lack of residues Asp 31 and Lys 47, along with Ser 9, was suggested to be linked to the lower toxicity of this protein [38,39]. Furthermore, the mechanism by which α-neurotoxins bind to their receptor (nAChR) and anchor to the membrane has been proposed to be based on multisite interaction [37]. It means that it could require specific regions from fingers I, II, and III for the proper interaction and activity [40,41,42,43,44]. For instance, the motif NQQSSQ in N-terminus region is highly conserved among α-neurotoxins [11,38,44,45,46,47] and it could be involved in the protein-binding to membranes. This hypothesis is exemplified by the importance of the amino acids at N-terminus of the NTII toxin for the interaction with membranes and activity on nAChR [44]. Furthermore, it has been suggested that the positively charged surface and the hydrophobic residues at the interface of the binding site in α-neurotoxins are also important to receptor recognition and binding [48], which is evident in the representative 3D structures of subgroup 1-A and 1-C (Figure S3).

**Figure 4:**
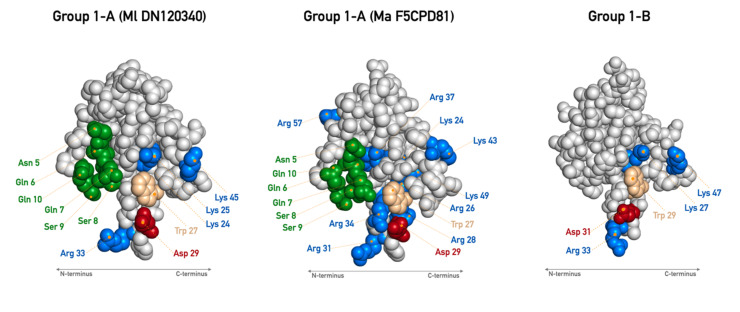

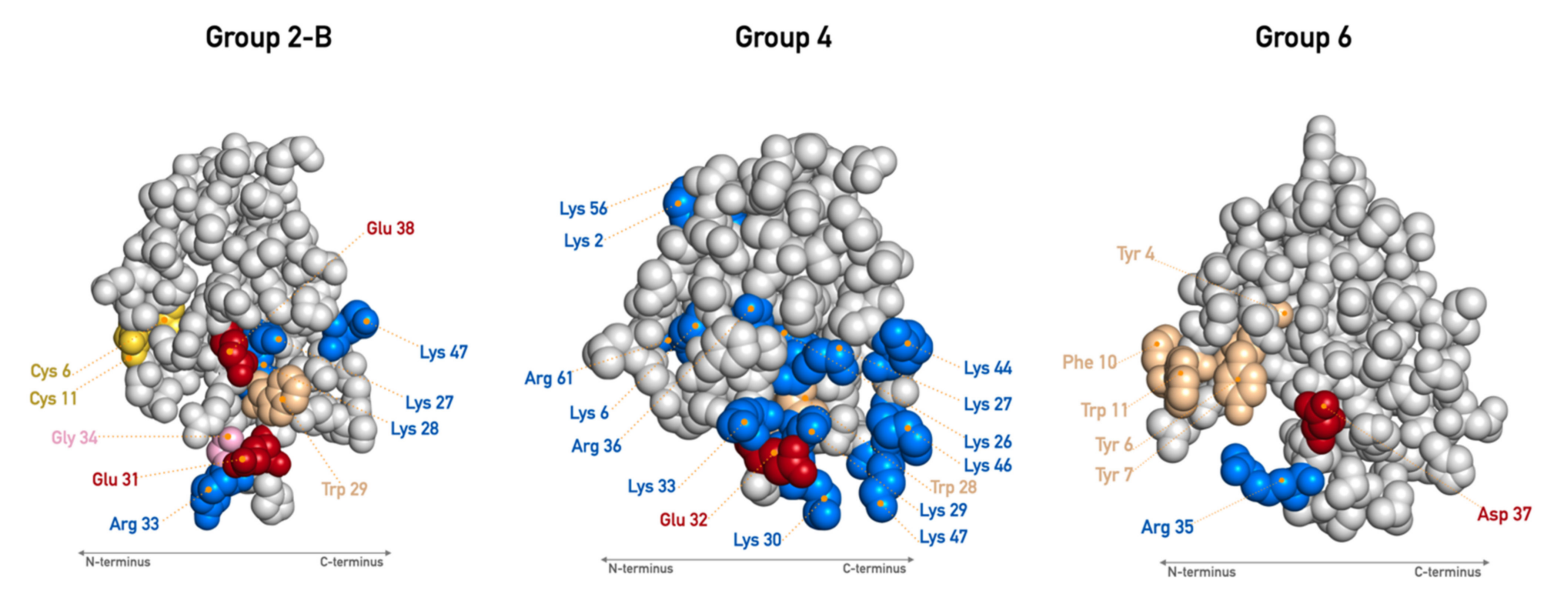
Functional amino acid residues and important regions in the representative 3FTxs of some groups. Some important/key amino acids discussed along the text are highlighted here in their corresponding groups. The coloring scheme is: red for negative amino acids, blue for positive, pale orange for aromatic resides, pale pink for glycine, yellow for cysteines, and green for the others. The grey arrows are indicating the orientation of the N and C-terminus. The fingers I, II, and III are positioned from the left to the right.

Subgroup 1-A has an α-neurotoxin (*Dendroaspis polylepis polylepis—*PDB 1NTX) [49] as structural homologue (Table S2). The sequences of this group have four disulfide bridges in the expected conserved positions, but one sequence (Ma AED895741) has an extra cysteine at position 55 in the C-terminus (Figure 1). Sequences from this group have the N-terminus very homogenous, similarly to classical α-neurotoxins. Some of the key amino acids for the activity on nAChR are present in these sequences, being Trp 27, Asp 29, Arg 31, and Lys 45, corresponding to the conserved residues mentioned for classical α-neurotoxins (Figure 1 and Figure 4). Moreover, they present a conserved double Lys residues pattern at positions 24 and 25 (Lys and Lys/Arg conserved similarly among groups 1 and 2), which may reflect in a more positive protein surface (Figure 1, Figure 4 and Figure S3). In fact, we can observe a similar charge surface distribution in this group compared to known α-neurotoxins (Figure S3). Also, the Tyr right after the third conserved structural Cys is very conserved among groups 1 and 2. Further, there are some sequences that outstand from others by having even more positive amino acids. This is the case of the Ma F5CPD81 sequence, which has Lys 24, Arg 26, Arg 28, Arg 31, Arg 34, Arg 37, Lys 43, and Lys 49, resulting in a very positive character at one of the protein face-side (Figure 4 and Figure S3). In general, subgroup 1-A is very homogeneous and very similar to its structural homologue, being a great candidate to be a group of post-synaptic short chain α-neurotoxin. Additionally, sequences from this group are grouped in a big cluster, branch 1, along with the other subgroups of group 1, in the phylogenetic analysis (Figure 3).

#### 3.1.2. Subgroup 1-B

Sequences from subgroup 1-B have the Atratoxin (PDB 1V6P) as their structural homologue (Tables S2 and S3), a short chain α-neurotoxin found in *Naja naja atra (mainland Chinese cobra)* venom with post-synaptic activity on nAChR [50,51]. Looking at the sequences from subgroup 1-B, they all have at the tip of the finger II the Arg residue. We also found Lys 27, Trp 29, Asp 31, and Lys 47 well conserved in almost all sequences of this group (Figure 1 and Figure 4). Interestingly, besides the presence of these key amino acids, the N-terminus (5 to 12 positions) of the sequences are markedly variable, in contrast to subgroup 1-A, which seems to not be common for short chain α-neurotoxins as already approached. Further analysis must be conducted to verify whether this relates to receptor binding specificity, or to cell membrane binding. Considering the positive charge surface as important for α-neurotoxins activity, as so, the Atratoxin has a quite positive protein surface. On the contrary, several negative amino acids are found in subgroup 1-B (e.g., positions 6, 15, and 26), which contribute greatly to a more negative potential at the surface of these proteins (Figure S3). In addition, all sequences in subgroup 1-B form a cluster when sorted out by phylogenetic analysis, with just the exception of Mp DN7425 sequence (Figure 3). In concert, these observations indicate that sequences from subgroup 1-B have important amino acids to exert function on nAChR as short chain α-neurotoxin, but some differences as in the N-terminus and charge surface may influence their activity. Also, these differences may direct these proteins to other functions, conferring the known multi-functionality present in 3FTxs.

#### 3.1.3. Subgroup 1-C

The structural homologue of subgroup 1-C is a toxin from Black mamba venom identified as MT9 protein (PDB 6F21—*Dendroaspis polylepis*) (Table S2). Unfortunately, this protein was not characterized and just the sequence and the crystal structure were reported. BLAST analysis showed that the closest protein to the MT9 is the PDB 1NTX (the homologue of subgroup 1-A), with only 52.54% of sequence identity. In summary, as the subgroup 1-C homologue is poorly understood, we could not compare information beyond their structure.

Similar to subgroups 1-A and B, sequences from subgroup 1-C are short chain neurotoxins. They have four conserved disulfide bridges and no extra cysteine. Comparing with subgroup 1-A and B, we noticed few similarities, since we found a very diversified N-terminus and finger II in subgroup 1-C. Nevertheless, we observed a similar structural motif composed by Gly, Cys, and Pro (GCGCP), which seems to be conserved among sequences from Group 1 and some α-neurotoxins (Figure 1). The common interaction site for curaremimetic activity, composed of aromatic and positively charged residues, was not clearly discerned in these sequences. However, the double Lys pattern (24 and 25 residues in the alignment positions) is present in almost all sequences, similar to subgroup 1-A. Noteworthy, we found an Asp 56 and a Lys 57 residues right before the last Cys conserved in all sequences of this subgroup. Similarly, we found the Asp 56 and Lys/Arg 57 in equivalent positions in almost all sequences within group 1 (Figure 1). Few aromatic residues are found, with only Tyr 23 (in the alignment position) conserved in all sequences (Figure 1). For the representative of the cluster (Mc Q9PUB71 sequence), its protein charge shows a predominant positive molecular surface (Figure S3), but it is difficult to generalize due to the considerable sequence variability. Finally, subgroup 1-C is also part of the phylogenetic branch 1 of candidates to be post-synaptic α-neurotoxin or alike (Figure 3).

#### 3.1.4. Subgroup 2-A

Subgroup 2-A is a small group composed of only two long chain sequences of 65 amino acids. Both sequences are distinguished by the presence of an extra disulfide bridge in finger I, different from other reported long chains where this extra bridge is present in finger II (Figure 1 and Figure 2). Its structural homologue is the γ-bungarotoxin (PDB 1MR6—*Bungarus multicinctus*) (Tables S2 and S3)*,* an RGD-containing protein with weak activity in inhibiting platelet aggregation [52]. Subgroup 2-A sequences do not have the Arg, Gly, and Asp residues (RGD motif) in the respective alignment position, having instead Arg, Gly, and Leu residues. Various positive and some hydrophobic residues are distributed along with the sequences, some of which may be related to interaction with nAChR (Figure 1). Regions with concentrations of positive and negative charges are present in the predicted protein electrostatic surface (Figure S3).

The already described 3FTxs with an extra disulfide bridge specifically in finger I, as well as the sequences from subgroup 2-A, are grouped in a class reported in the literature as “Weak neurotoxins”. Lately, this class has been recognized as non-conventional toxins. Toxins from this group typically have lower toxicity than other long chains. Non-conventional toxins may present their toxicity about LD50 of 5–80 mg/kg, in opposition to prototype α-neurotoxins, which may present LD50 of 0.04–0.3 mg/kg [53]. Exceptions to this knowledge exist, and this is why the term “Weak neurotoxin” is falling into disuse. For example, the γ-bungarotoxin, structurally similar to “Weak neurotoxins”, has an LD50 of 0.15 mg/kg, which is more related to α-neurotoxins activity [52,54]. Despite the reported common lower toxicity of the non-conventional toxins, they could present variable activities that are poorly explored to date [53]. Additionally, in a mutagenesis study of a weak neurotoxin from *Naja kaothia*, the content of arginine in finger II was demonstrated to be essential for interaction on M1, M2, and M3 muscarinic AChR (mAChR) [55]. Those Arg residues are also observed in sequences from subgroup 2-A and B (Figure 1 and Figure 4). In the phylogenetic analysis, both sequences from subgroup 2-A are settled together in the same big branch as group 1 (Figure 3).

#### 3.1.5. Subgroup 2-B

The subgroup 2-B structural homologue is the 3FTx called Candoxin (PDB 1JGK) (Tables S2 and S3), from *Bungarus candidus* venom. This toxin was considered a novel long chain 3FTx, because of its reversible antagonistic effect on muscle-type and weaker but also on neuronal α7 nAChR. In other similar toxins, the activity is commonly almost or totally irreversible [56]. As in the subgroup 2-A, sequences from subgroup 2-B are differentiated by the presence of a fifth extra disulfide bridge located in the finger I, as found in Candoxin. These sequences are long chains with smaller C-terminus tails, similar to Candoxin but different from almost all other long chain neurotoxins. As in group 1, they have some of the key amino acids for the activity on nAChR. We found Lys/Arg 27, Trp 29, and Glu/Asp 31 conserved in all sequences. The notorious Arg 33 is present only in one sequence (Mf P864231), while the others have instead a His, Gln, or Asn. Additionally, Gly 34 and Glu 38, critical for erabutoxin-a [57] and found in Candoxin, are also present in most of the members of subgroup 2-B (Figure 1 and Figure 4). Some of those residues are common in some neurotoxins and are pointed out as important both for activity on muscle and on neuronal nAChR. Moreover, the structural homologue of subgroup 2-B, Candoxin, lacks the helix-like conformation at the tip of the finger II, which is present in the α-cobratoxin and is related to high affinity to α7 receptor [58]. Altogether, this could suggest that the sequences from subgroup 2-B can compose a new class of 3FTxs along with Candoxin. In the phylogenetic analysis, this group is also part of branch 1 (Figure 3).

#### 3.1.6. Group 3

Group 3 has Bucain (PDB 2H8U) as structural homologue (Tables S2 and S3), a 3FTx from the Malayan Krait *Bungarus candidus* characterized as a potent neurotoxin and structurally similar with α-neurotoxins, with a positively charged AChR-binding site [48]. Even sharing the Bucain as structural homologue, sequences in this group present some important differences. Some of these are the changes of the amino acids Asp/Leu 4, Asp/Ile 5, Arg/Glu 12, -/Lys 26, Val or Ile/Trp 28, Tyr/Gly 35, and Glu/Lys 36 in the alignment positions of group 3 and Bucain, respectively (Figure 1). Some of these involve drastic changes as in aromatic, positive, and negative amino acids, certainly driving these proteins to different activities or affinity to receptors. The electrostatic prediction demonstrated the homogeneous distribution of positive and negative charges on the protein surface (Figure S3). Further, the sequences of this group were gathered in branch 3 in the phylogenetic tree (Figure 3).

#### 3.1.7. Group 4

Group 4 (homologue PDB 3HH7, from *Ophiophagus hannah* venom) (Tables S2 and S3), stands out from the other groups by having an astonishing content of positive amino acids among its sequences. This characteristic caught our attention as it could suggest direct interactions with membranes, perhaps as cardiotoxin/cytotoxins. We can observe at least 12 positive residues highly conserved among the group 4 sequences, almost all being lysines concentrated in fingers II and III (Figure 1 and Figure 4). This reflects in a distinguished positively charged protein surface, as predicted for the representative sequence, and that can be extended for the others (Figure S3).

The group 4 structural homologue is named Haditoxin. This protein was described as a novel neurotoxin because of its pharmacological activity, an antagonism towards both muscle and neuronal nAChR. It was reported as the first dimeric short chain α-neurotoxin with activity on α7 nAChR and also as the first homodimeric 3FTx with activity on muscle-type nAChR [59]. Notably, group 4 has important differences mainly regarding the presence of the lysines in important positions, when compared to Haditoxin. This probably directs these proteins to other functions, and our analyses suggest that this group may be closely related to cardiotoxin/cytotoxin. In the phylogenetic analysis, group 4 is in branch 2, grouped with Bucain, the structural homologue of group 3 (Figure 3).

#### 3.1.8. Group 5

Group 5 (homologue PDB 2LA1, from *Dendroaspis jamesoni kaimosae*) (Table S2 and S3) is a small group with only two sequences. This group has a very low sequence identity, which reflects in a difficulty to predict a three-dimensional structure, even having a structural homologue (Figure 1). As shown in Figure 2, the representative 3D model seems very flexible in each finger, with predominance of random like-loop conformation. The prediction of secondary structure by JPred server proposed a β-stranded pattern in fingers II and III, not corroborating with our structural model (Figure S2). We observed only 14 amino acids conserved in the alignment, which includes the cysteines. Due to the low identity, we could not identify key residues in those sequences to have clues about their function. Moreover, we can not expect a good accuracy for the structural prediction in this group. This group is peculiar and probably has different activities, perhaps never identified in other 3FTxs around the world. Dendroaspin, the homologous protein of group 5, is a 3FTx containing a PRGDMP motif with potent activity on inhibiting integrins [60]. Sequences from group 5 do not have this motif and neither the RGD. Not surprisingly, the phylogenetic analysis placed each sequence from group 5 in two different branches (4 and 5) (Figure 3).

#### 3.1.9. Group 6

Group 6 has considerable sequence homogeneity. Even though, comparing this group with its homologue, a 3FTx called Ringhalexin (PDB 4ZQY—from *Hemachatus haemachatus*) (Tables S2 and S3), we can observe low sequence identity mainly in finger I and II (Figure 1). One interesting characteristic observed in these sequences is a great content of aromatic residues in the N-terminal portion (Tyr 4, Tyr 6, Tyr 7, Phe 10, Trp 11), not fully conserved in the homologous nor in the sequences of the other groups studied here (Figure 1). The presence of aromatic amino acids at the protein surface is frequently related to protein–protein interaction such as in dimerization interfaces [61,62].

Regarding the structural homologue, Ringhalexin, in addition to its weak and irreversible neurotoxicity, it shows potent anticoagulant property through the inhibition of extrinsic tenase complex, which comprises the tissue factor—factor VIIa (TF-FVIIa) [63]. In a docking study performed with this toxin and two other 3FTxs with high identity and similarity to Ringhalexin, there were proposed twelve amino acids that may contribute to the specificity of these proteins towards the TF-FVIIa, being the Tyr 7, Lys 9, Glu 12, Lys 26, Arg 34, Leu 35, Arg 40, Val 55, Asp 56, Cys 57, Cys 58, and Arg 65 [64]. Looking at group 6, we found Tyr 7, Lys 26, Arg 40, Val/Leu 55, Glu 56 instead of Asp, Cys 57, and Cys 58 amino acids (Figure 1). In the phylogenetic tree, all sequences were grouped well in branch 2 (Figure 3).

#### 3.1.10. Group 7

Group 7 is one of the most homogenous groups of toxins from this study with high sequence identity (Figure 1). The structural homologue is the Bucandin (PDB 1F94—from *Bungarus candidus* species) (Tables S2 and S3), a 3FTx with a unique property of enhancing the release of acetylcholine, acting pre-synaptically [65]. To the best of our knowledge, this is the only 3FTx described to date with pre-synaptic activity. Just like group 2, sequences from group 7 have an extra disulfide bond in finger I, which sets these sequences as non-conventional according to the literature. In Bucandin, the extra fifth bond causes an unusual kink in finger I that twists this region away from the rest of the protein. It was speculated that this structure could be a useful way to isolate this region to prevent charge interference or to rigidify the molecule to lead to some specific interaction with a target [65,66]. Considering the high sequence identity of group 7 with Bucandin, especially in the first finger, we believe that this structure may also occur in this group. In the 3D model, we could observe a similar structure also in the first finger, which seems to be directed also by the extra disulfide bond (Figure 2). Curiously, the JPred software predicted two α-helices in finger II, whereas in our model a β-sheet is found instead (Figure 2 and Figure S2). To note, no 3FTx with the structure already determined, including the Bucandin, present α-helices. Moreover, there is more positive charge distributed throughout the protein than negative, according to the prediction of electrostatic surface (Figure S3). In Bucandin we can observe two distinct charged faces, conferring an amphipathic character to the molecule [65,66]. In respect to the phylogenetic analysis, group 7 sequences and Bucandin are clustered together in branch 4, reinforcing the great proximity in the perspective of both structure and phylogenetic relationship (Figure 3).

#### 3.1.11. Group 8

Group 8, which comprises subgroups 8-A and 8-B, represents one of the most variable groups, with low sequence identity among their ten members. The principal reason why these two groups were gathered is a structural characteristic, an extension of finger II in relation to the other fingers observed only in this group. Interestingly, subgroup 8-A has the Mambalgin (from *Dendroaspis polylepis*, PDB 5DO6) as structural homologue, while 8-B has the Fulditoxin (from *Micrurus fulvius*, PDB 4RUD) (Tables S2 and S3), two different toxins having distinct functional roles. Even though, the similarity among these 3D structures is notable, with few differences mainly in the outermost section of fingers I and II. Mambalgin interacts with acid sensing ion channel causing an effect of abolishing pain [16,67]. In contrast, Fulditoxin is a new short-chain α-neurotoxin that forms a homodimer by hydrophobic contacts and also is the first 3FTx ever described that binds metal and has the ability to assemble tetrameric complexes. Its activity is a reversible post-synaptic neuromuscular blockade through the nAChR [68]. Even if some of the key functional amino acids described for the homologous could be found in some of the sequences of group 8, its sequence variability suggests novel roles that should be further studied. In the phylogenetic analyses, subgroups 8-A and B are clustered in branch 5 (Figure 3).

#### 3.1.12. Group 9

The group 9 structural homologue is a cardiotoxin from *Naja atra* venom called CTX A5 (PDB 1KXI) [69] (Tables S2 and S3). Even with important differences in the primary structure (Figure 1), both sequences from group 9 are disposed in the same cluster (branch 5) one next to the other in the phylogenetic tree (Figure 3). Comparing group 9 with its homologue, we could not predict beyond the overall 3D structure (Figure 2).

### 3.2. Undiscovered Biotechnological Potential of 3FTxs

In the past years, the interest as well as the knowledge concerning the 3FTxs found in *Elapid* venoms throughout the world have been growing. In fact, this is happening also because of a trend in toxinology to study, in particular, small toxins present in venoms of many different types of animals (*e.g.,* sea anemones, cone snails, scorpions, spiders, snakes), which are capable of specifically interfering with a range of channels/receptors in animal organisms [70,71,72]. In the case of 3FTxs, the mentioned trend was probably amplified by the discoveries of new functions in the last decade, as the Mambalgin activity, for example [16,73]. Naturally, several very interesting functions for these toxins contribute and are still raising interest in the field of biodiscovery, biotechnology, and pharmacology. In general, the growing knowledge of the animal-toxins biotechnological potential is causing a far-reaching interest among researchers inside and outside of the toxinology community.

Due to their small size and multi-functionality convened in a diversified primary structure, the 3FTx family is also of biotechnology interest. In Brazil, the knowledge regarding the 3FTxs from Elapid species is still poor and underestimated. Part of this is caused due to the interpretation of the toxin’s activity, based mostly on general clinical patterns of the envenomation. However, as we know, lots of different activities can coexist in the same toxin family and even in the same particular 3FTx. Although, one specific activity may be masked by the presence of the most abundant proteins in the venom, which warrants studies like this that may lead to the identification of novel activities. Another important factor that contributes to the lack of knowledge is the difficulty in obtaining a reasonable amount of venom to study. In this way, the tracking of new molecules must be undertaken to amplify the comprehension of these toxins in Brazil and to increase the availability of proteins with biotechnological potential.

## 4. Conclusions

In this work, we presented some 3FTxs from Brazilian *Micrurus* species and classified them mostly based on structural features. We are showing a new way to cluster toxins with distinguished variability in primary structure using the developed guideline. Comparing the 3FTx groups with their homologous proteins, we were able to point important/key amino acids and structural characteristics related to the activity. Moreover, we found some sequences with very low identity with any other characterized 3FTx, suggesting novel roles for those toxins.

Since the most described functions of 3FTxs from *Micrurus* venoms are on cholinergic receptors, in this study we could give more insights related to this activity. For all other activities described for the 3FTx family, more studies must be undertaken to find frequent regions related to the activity for those infrequent and particular functions. As so, taking into consideration the well-recognized diversity inside the 3FTx family and the vast universe of unexplored structures and functions, in this work we approached just the tip of the possibilities behind this unique group of toxins. We hope that the present work enriches discussions and instigates research about the 3FTxs from *Micrurus* venoms and for other groups of toxins.

## 5. Methods

### 5.1. Sequence Database

In a first step, a non-curated dataset was constructed by joining 3FTxs sequences published in Aird et al., 2017 [3] and sequences from GenBank (NCBI), all described as from *Micrurus* species from Brazil. After manually removing truncated sequences (e.g., presenting less than eight cysteines), and those with 100% identity (redundant), the final curated dataset comprises 57 3FTxs sequences, being 28 sequences of 3FTxs published in Aird et al., 2017 [3] and 29 sequences retrieved from GenBank (NCBI) (see Table S1).

### 5.2. Toxin Classification Based on Homology Predictions

Due to the low sequence identity in this toxin family (Figure S1), structural-homologous proteins were searched by an algorithm based on profile hidden Markov models (HMMs), which is especially useful for remote protein homology detection [30,31]. As our objective was to perform afterwards a structural prediction and analysis of those sequences, the HHPred server [32,33], available online in Bioinformatics Toolkit (https://toolkit.tuebingen.mpg.de/tools, accessed on 29 April 2021), was selected to identify the best structural homologous template by using HMM-HMM comparison. The sequences in our database were first classified based on the best hit (lowest E-value) given by HHPred for each of them. In the cases when HHPred was giving similar or identical E-value for several PDB (Protein Data Bank) entries belonging to different proteins, all of them were inspected manually and, afterward, other analyses (e.g., disulfide bond pattern, primary structure signatures) were considered to choose the best homologue (see Table S2).

### 5.3. Homology Model Building

As we grouped proteins that had the same predicted structural homologue, we expected them to present a similar fold, and we selected therefore just one protein from each group as a representative for model building. To build a three-dimensional model, we used, as template, the PDB entry suggested by HHPred software and also the sequence alignment between target and template given by the server. An exception was group 5 (homologous PDB 2LA1), in which the sequence alignment proposed by HHPred did not pair one of the conserved cysteines and it was manually modified to adjust to the same position as in the structural homologous. The Modeller software [74] (9.20 version, University of California San Francisco, San Francisco, CA, USA) was used to build 100 models per sequence. We selected as the most probable structural model the one with the lowest DOPE (discrete optimized protein energy) score [75]. The models were visualized in PyMOL Molecular Graphics System, version 2.2 Schrödinger, LLC, Cambridge, UK. Additionally, the secondary structure of the representative protein of each group was predicted with the JPred server. This server is based on the Jnet algorithm, a two-level neural network algorithm trained using the aligned sequences of 480 proteins. Also, it calculates solvent accessibility and coiled-coil regions using the Lupas method [76]. This independent analysis was useful to check whether or not those predictions matched with the secondary structure proposed by our three-dimensional models constructed by the Modeller software.

### 5.4. Analysis of Primary Structure and Sequence Alignment

Sequence alignment was performed using the MUSCLE algorithm [77] in the Molecular Evolutionary Genetics Analysis (MEGA) software [78]. The given alignments were visualized with the Jalview software [34] version 2.10.5, open source bioinformatics software, Dundee, Scotland, UK.

### 5.5. Electrostatic Surface Potential Calculation

The calculation of the electrostatic surface potential of both the three-dimensional models and their PDB templates was performed with the Adaptive Poisson-Boltzmann Solver (APBS) tool, implemented as a plugin in the PyMol software. The default parameter setting was used for the analysis and the results were expressed in kT unit (Boltzmann constant/temperature).

### 5.6. Phylogenic Analysis

The evolutionary relationship was inferred by using the maximum likelihood analysis performed by the Molecular Evolutionary Genetics Analysis (MEGA) software. In order to find the best matrix of substitution, we tested automatically 56 different models. We chose the Whelan And Goldman (WAG) and the Gamma distributed with Invariant sites (G+I) as substitution model and as the evolutionary rate differences among sites, respectively, based on the lower value of the BIC score provided by MEGA software. To construct the initial tree for the heuristic search, we automatically applied the Neighbour-Join and BioNJ algorithms to a matrix of pairwise distances estimated by using the JTT model. The tree nodes were supported by 1000 bootstrapping replicates. The tree topology with superior log likelihood value was selected. 80 sequences were used for this analysis, being: 57 sequences of our dataset of 3FTx from *Micrurus* from Brazil (Table S1), 13 sequences from the structural homologues used in the present work, and 10 sequences of other 3FTxs already characterized in the literature. All positions containing gaps and missing data were eliminated.

### 5.7. Group Refinement and Biological Insights Annotation

The group’s refinement and a hypothesis about the biological activity and the molecular properties of each cluster of toxins were suggested based on (1) the biological activity and structure of their proposed structural homologue, if known; (2) sequence analysis of conserved regions and disulfide bridges pattern; (3) phylogenetic relationship; (4) calculated charge distribution on the protein surface. Using this analysis, some of our primary groups were further banded together and proposed as subgroups (e.g., group 1). With this information in hand, we managed to propose a structure-function classification of 3FTxs into 9 main groups (Figure 1).

## Data Availability

The data presented in this study are openly available in Zenodo at http://doi.org/10.5281/zenodo.4728151, accessed on 29 April 2021.

## Supplementary Materials

The following are available online at https://www.mdpi.com/article/10.3390/toxins13050328/s1, Figure S1: Secondary structure prediction for each representative model for each 3FTx groups; Figure S2: APBS electrostatic potential surface for each representative structure for each group; Figure S3: Three-finger toxin sequences from Brazilian Micrurus species; Table S1: Sequence Identifier; Table S2: Selected structural homologues suggested by the HHPred server and curated manually; Table S3 Survey of some members of the 3FT family already characterized.
