# Supplementary material for: Three-Finger Toxins from Brazilian Coral Snakes: From Molecular Framework to Insights in Biological Function
Source: Toxins (Basel). 2021 Apr 30;13(5):328. doi: 10.3390/toxins13050328 (PMC8147190; doi:10.3390/toxins13050328)
Supplement: Supplementary file 1 [file toxins-13-00328-s001.zip › toxins-1197849-supplementary.pdf]

Supplementary Material: Three-Finger Toxins from Brazilian Coral Snakes: From Molecular Framework to Insights in Biological Function

Jessica Matos Kleiz-Ferreira, Nuria Cirauqui, Edson Araujo Trajano, Marcius da Silva Almeida and Russolina Benedeta Zingali

SUBGROUP 1-A

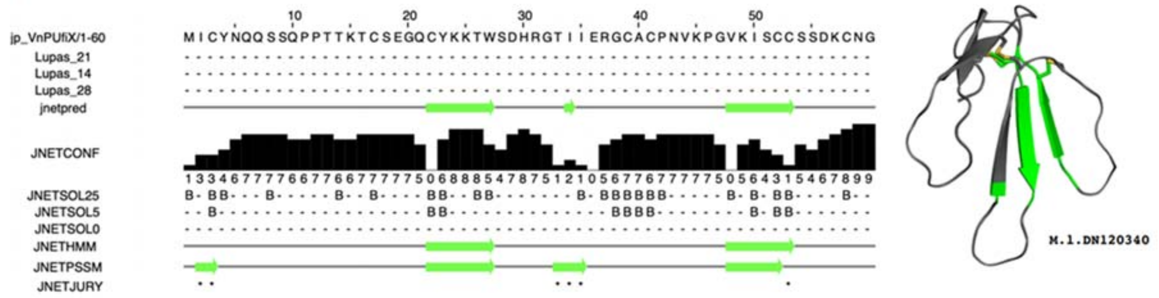

SUBGROUP 1-B

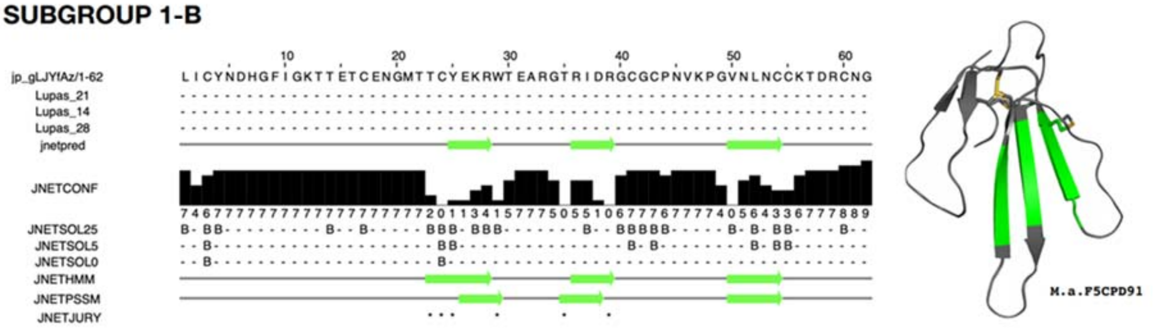

SUBGROUP 1-C

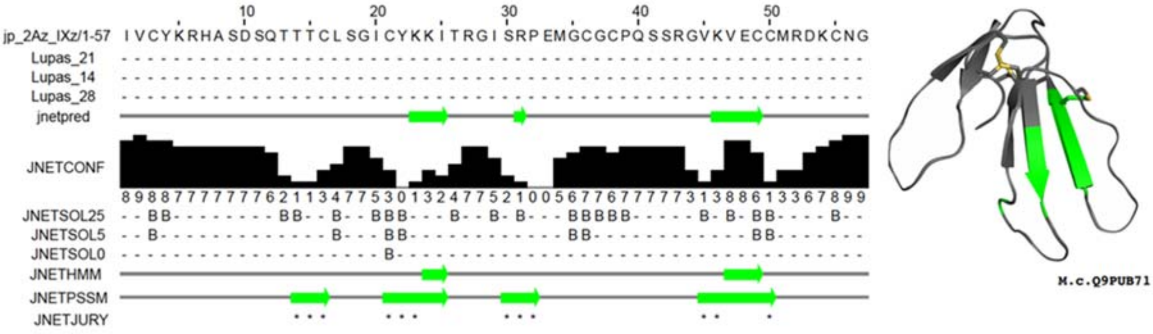

SUBGROUP 2-A

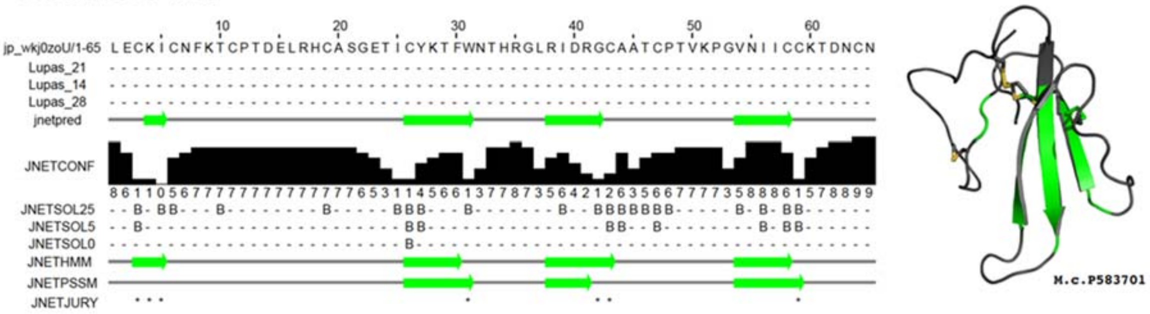

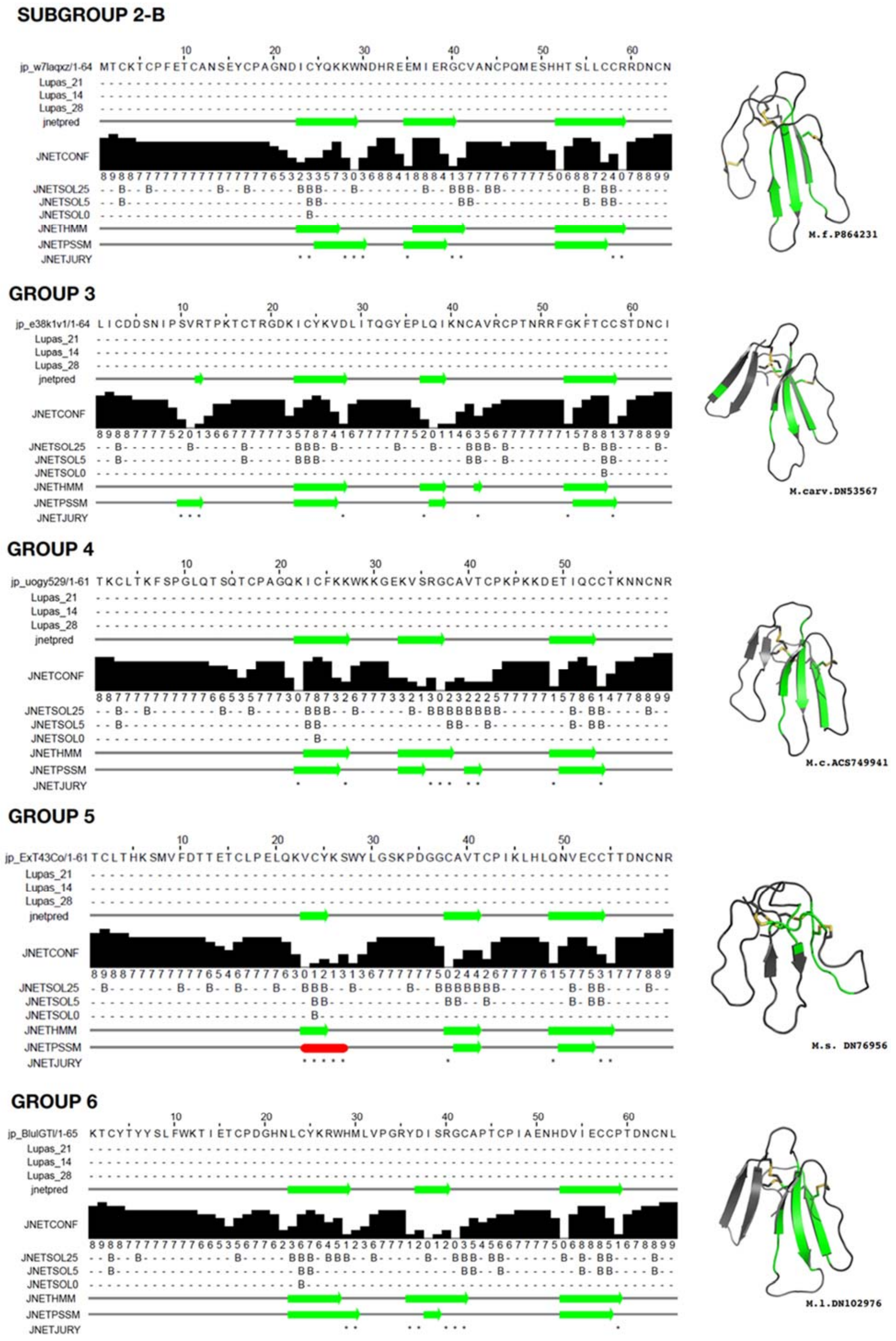
SUBGROUP 2-B
jp_w7laqxz/1-64 MTCKTCPFETCANSEYCPAGNDICYQKKWNDHREEMIERGCVANCPQMESHHTSLLCCRRDNCN
M.f.P864231
GROUP 3
jp_e38k1v1/1-64 LICDDSNIPSVRTPKTCTRGDKICYKVDLITQGYEPLQIKNCAVRCPTNRRFGKFTCCSTDNCI
M.carv.DN53567
GROUP 4
jp_uogy529/1-61 TKCLTKFSPGLQTSQTCPAGQKICFKKWKKGEKVSRGCAVTCPKPKKDETIQCCTKNNCNR
M.c.ACS749941
GROUP 5
jp_ExT43Co/1-61 TCLTHKSMVFDTTETCLPELQKVCYKSWYLGSKPDGGCAVTCPIKLHLQNVECCTTDNCNR
M.s. DN76956
GROUP 6
jp_BluIGTI/1-65 KTCYTYYSLFWKTIETCPDGHNLCYKRWHMLVPGRYDISRGCAPTCPIAENHDVIECCPTDNCNL
M.l.DN102976
Lupas_21
Lupas_14
Lupas_28
jnetpred
JNETCONF
JNETSOL25
JNETSOL5
JNETSOL0
JNETHMM
JNETPSSM
JNETJURY

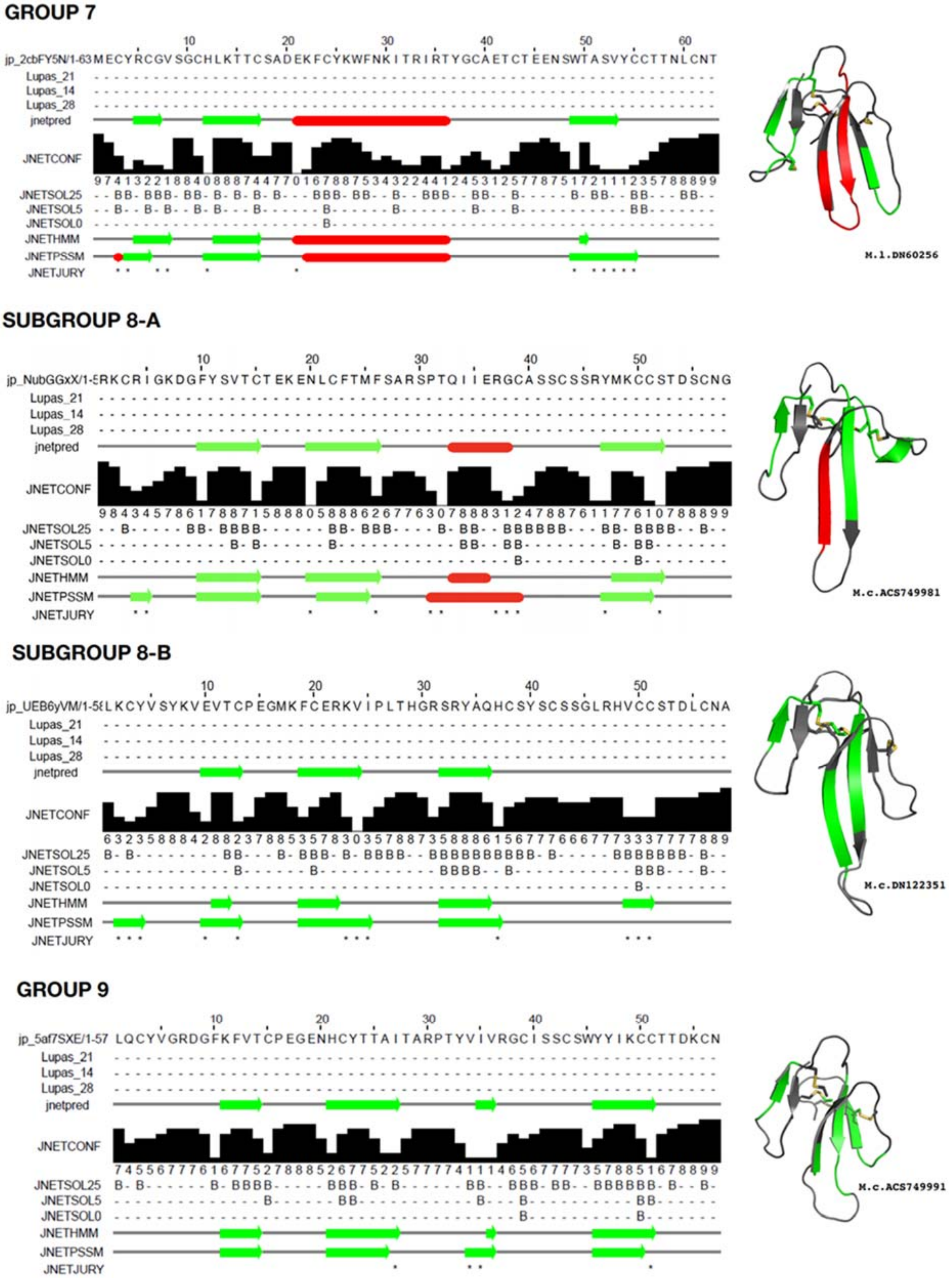

Figure S1. Secondary structure prediction for each representative model for each 3FTx groups. For each group, the results of different algorithms for secondary structure prediction in the JPred software are shown. In the right, we can see the model of the representative of each group colored according to the consensus prediction (jnetpred). The confidence of the prediction is indicated by the JNetConf scale, from 0 to 9. Green color both in the alignment and the structure represents beta sheets. Red color represents alpha helix.

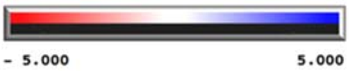

GROUP 1

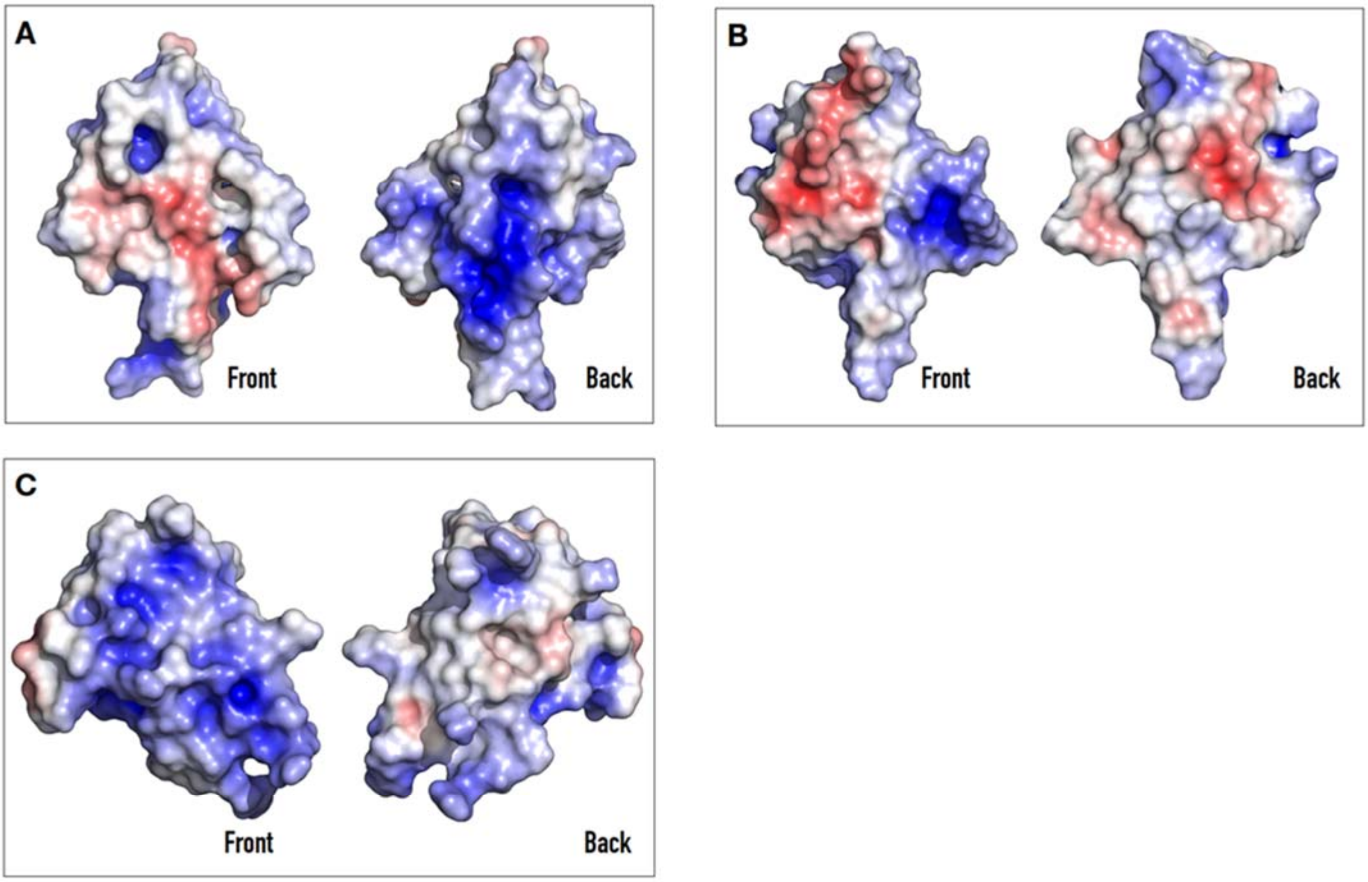

GROUP 2

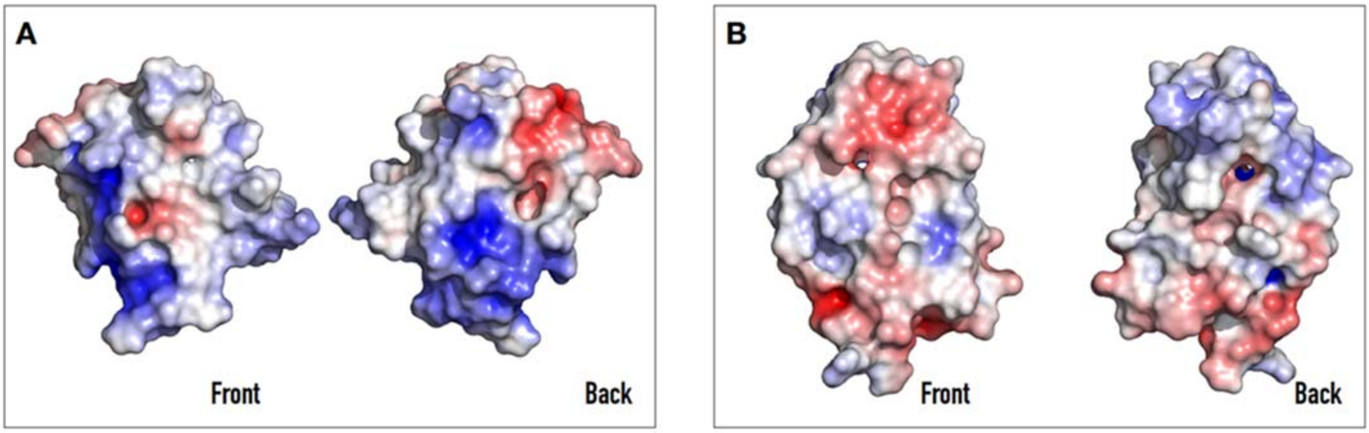

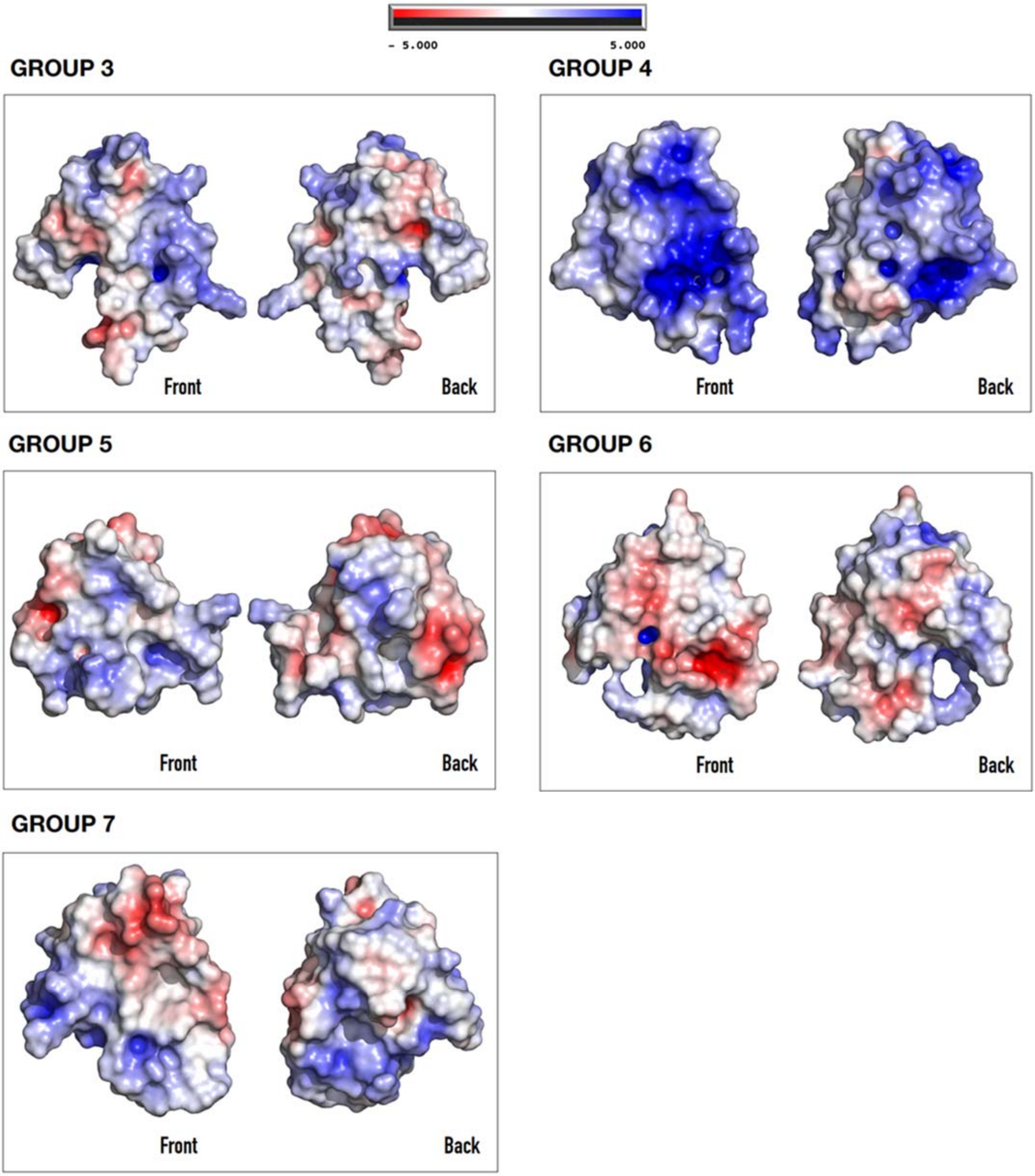
- 5.000
5.000
GROUP 3
Front
Back
GROUP 4
Front
Back
GROUP 5
Front
Back
GROUP 6
Front
Back
GROUP 7
Front
Back

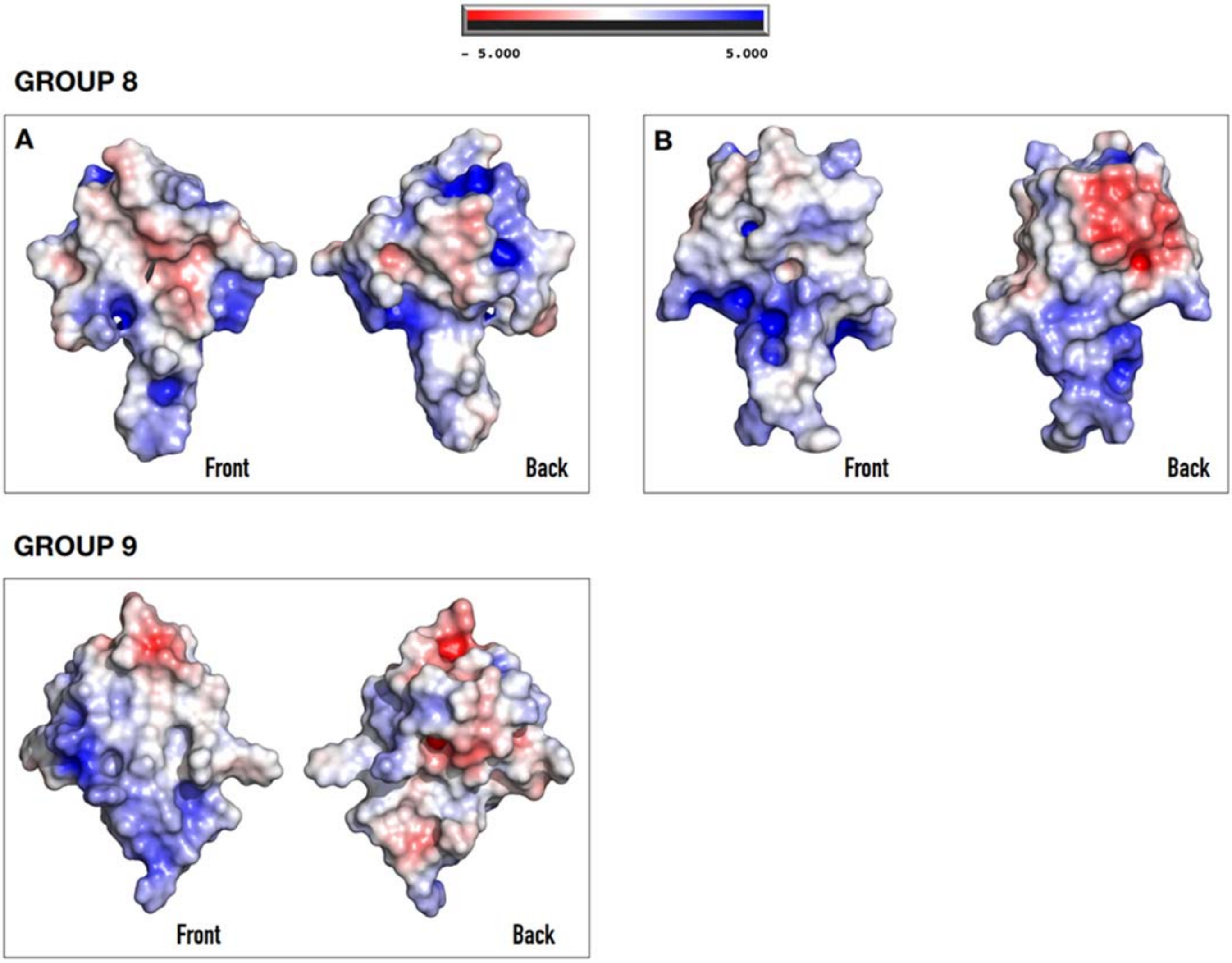

Figure S2. APBS electrostatic potential surface for each representative structure for each group. The electrostatic potential is represented by +/- 5 kT/e, being blue color (positive charge), red color (negative charge) and white color (neutral charge).

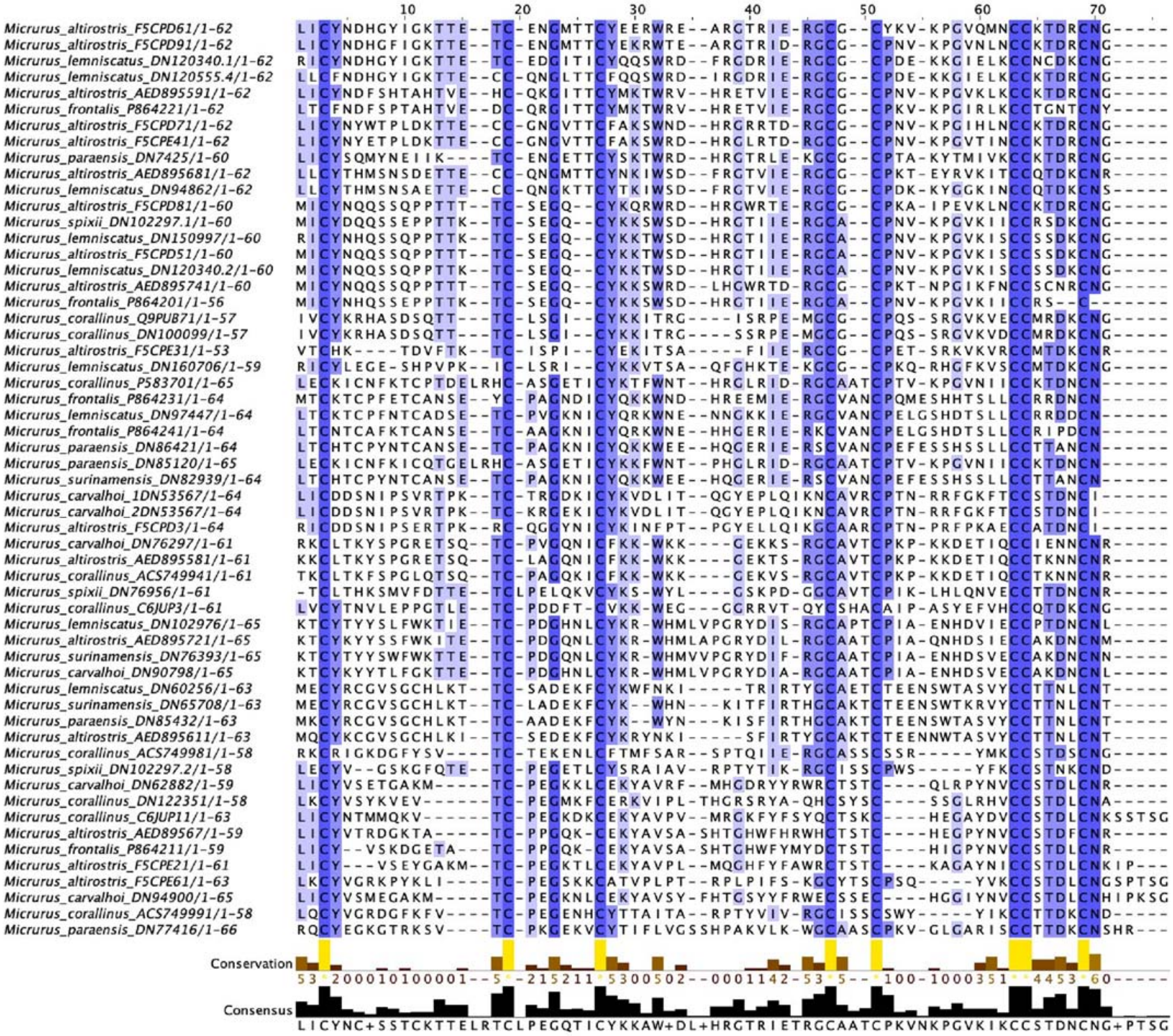

Figure S3. Three-finger toxin sequences from Brazilian Micrurus species. Global alignment of the 3FTx sequences used in the present study. Conservation is shown by purple with the more conserved regions shadowed darker. The figure was created in Jalview and colored with the Percentage identity scheme.

Table S1. Sequence Identifier. 3FTx sequences from Brazilian Micrurus used in this work, classified according to the groups in Figure 1. The database from which the sequences were retrieved is indicated, together with its code in the database and the reference of the work that published the sequence.

	Species	Code	Databank	Reference
**Subgroup 1-A**	*Micrurus altirostris*	F5CPD8.1	NCBI	[1]
	Micrurus altirostris	AED89574.1	NCBI	[1]
	Micrurus altirostris	F5CPD5.1	NCBI	[1]
	Micrurus spixii	DN102297/m.10839	Personal database	[2]
	Micrurus frontalis	P86420.1	NCBI	[3]
	Micrurus lemniscatus	DN120340.2/m.46961	Personal database	[2]
	Micrurus lemniscatus	DN150997	Personal database	[2]
**Subgroup 1-B**	*Micrurus altirostris*	F5CPD6	NCBI	[1]
	Micrurus altirostris	F5CPD9	NCBI	[1]
	Micrurus altirostris	AED89559.1	NCBI	[1]
	Micrurus altirostris	F5CPD7	NCBI	[1]
	Micrurus altirostris	F5CPE4.1	NCBI	[1]
	Micrurus altirostris	AED89568.1	NCBI	[1]
	Micrurus lemniscatus	DN120340.1/m.46960	Personal database	[2]
	Micrurus lemniscatus	DN120555/m.34930	Personal database	[2]
	Micrurus lemniscatus	DN94862	Personal database	[2]
	Micrurus frontalis	P86422.1	NCBI	[4]
	Micrurus paraensis	DN7425	Personal database	[2]
**Subgroup 1-C**	*Micrurus corallinus*	Q9PUB7.1	NCBI	[5]
	Micrurus corallinus	DN100099	Personal database	[2]
	Micrurus altirostris	F5CPE3.1	NCBI	[1]
	Micrurus lemniscatus	DN160706	Personal database	[2]
**Subgroup 2-A**	*Micrurus corallinus*	P58370.1	NCBI	[1]
	Micrurus paraensis	DN85120	Personal database	[2]
**Subgroup 2-B**	*Micrurus frontalis*	P86423.1	NCBI	[4]
	Micrurus frontalis	P86424.1	NCBI	[4]
	Micrurus surinamensis	DN82939	Personal database	[2]
	Micrurus lemniscatus	DN97447	Personal database	[2]
	Micrurus paraensis	DN86421	Personal database	[2]
**Group 3**	*Micrurus carvalhoi*	DN53567.1	Personal database	[2]
	Micrurus carvalhoi	DN53567.2	Personal database	[2]
	Micrurus altirostris	F5CPD3	NCBI	[1]
**Group 4**	*Micrurus carvalhoi*	DN76297	Personal database	[2]
	Micrurus altirostris	AED89558.1	NCBI	[1]
	Micrurus corallinus	ACS74994.1	NCBI	[6]
**Group 5**	*Micrurus spixii*	DN76956	Personal database	[2]
	Micrurus corallinus	C6JUP3	NCBI	[6]
**Group 6**	*Micrurus lemniscatus*	DN102976	Personal database	[2]
	Micrurus altirostris	AED89572.1	NCBI	[1]
	Micrurus surinamensis	DN76393	Personal database	[2]
	Micrurus carvalhoi	DN90798	Personal database	[2]
**Group 7**	*Micrurus lemniscatus*	DN60256	Personal database	[2]

	Micrurus surinamensis	DN65708	Personal database	[2]
	Micrurus paraensis	DN85432	Personal database	[2]
	Micrurus altirostris	AED89561	NCBI	[1]
**Subgroup 8-A**	*Micrurus corallinus*	ACS74998.1	NCBI	[6]
	Micrurus spixii	DN102297/m.10825	Personal database	[2]
	Micrurus carvalhoi	DN62882	Personal database	[2]
**Subgroup 8-B**	*Micrurus corallinus*	DN122351	Personal database	[2]
	Micrurus corallinus	C6JUP1	NCBI	[6]
	Micrurus altirostris	AED89567	NCBI	[1]
	Micrurus altirostris	F5CPE2	NCBI	[1]
	Micrurus altirostris	F5CPE6	NCBI	[1]
	Micrurus frontalis	P86421	NCBI	[4]
	Micrurus carvalhoi	DN94900	Personal database	[2]
**Group 9**	*Micrurus corallinus*	ACS74999	NCBI	[6]
	Micrurus paraensis	DN77416	Personal database	[2]

Table S2. Selected structural homologues suggested by the HHPred server and curated manually. For each sequence, the suggested homologues were considered after manual curation. The groups, species, sequence code, the final selected structural homologue and the corresponding E-values are shown.

	Species	Sequence code	Selected structural homologue predicted by HHPred (PDB code)	E-value
**Subgroup 1-A**	*Micrurus altirostris*	F5CPD8.1 *	1NTX	2.5e-18
	Micrurus altirostris	AED89574.1 *		3.1e-18
	Micrurus altirostris	F5CPD5.1		3.6e-18
	Micrurus spixii	DN102297/m.10839		2.7e-18
	Micrurus frontalis	P86420.1 *		9.4e-15
	Micrurus lemniscatus	DN120340.2/m.46961		3.1e-18
	Micrurus lemniscatus	DN150997		2.4e-18
**Subgroup 1-B**	*Micrurus altirostris*	F5CPD6	1V6P	2.3e-18
	Micrurus altirostris	F5CPD9		3.8e-19
	Micrurus altirostris	AED89559.1		1.0e-18
	Micrurus altirostris	F5CPD7		5.2e-19
	Micrurus altirostris	F5CPE4.1		1.2e-18
	Micrurus altirostris	AED89568.1		2.2e-18
	Micrurus lemniscatus	DN120340.1/m.46960		6.5e-19
	Micrurus lemniscatus	DN120555/m.34930		8.3e-19
	Micrurus lemniscatus	DN94862		5.4e-18
	Micrurus frontalis	P86422.1		1.2e-18
	Micrurus paraensis	DN7425		3.5e-17
**Subgroup 1-C**	*Micrurus corallinus*	Q9PUB7.1	6F21	5.4e-17
	Micrurus corallinus	DN100099		1.3e-17
	Micrurus altirostris	F5CPE3.1		3.7e-17
	Micrurus lemniscatus	DN160706		2.9e-18
**Subgroup 2-A**	*Micrurus corallinus*	P58370.1 *	1MR6	3.6e-17
	Micrurus paraensis	DN85120		1.1e-17
**Subgroup 2-B**	*Micrurus frontalis*	P86423.1	1JGK	6.7e-19
	Micrurus frontalis	P86424.1 *		1.8e-18
	Micrurus surinamensis	DN82939 *		1.5e-18
	Micrurus lemniscatus	DN97447 *		1.6e-18
	Micrurus paraensis	DN86421 *		1.5e-18
**Group 3**	*Micrurus carvalhoi*	DN53567.1	2H8U	3.1e-19
	Micrurus carvalhoi	DN53567.2		5.4e-19
	Micrurus altirostris	F5CPD3 *		6.7e-19
**Group 4**	*Micrurus carvalhoi*	DN76297 *	3HH7	7.0e-17
	Micrurus altirostris	AED89558.1		3.2e-17
	Micrurus corallinus	ACS74994.1		9.8e-17
**Group 5**	*Micrurus spixii*	DN76956	2LA1	6.9e-17
	Micrurus corallinus	C6JUP3		9.6e-19
**Group 6**	*Micrurus lemniscatus*	DN102976 *	4ZQY	2.3e-18
	Micrurus altirostris	AED89572.1		2.3e-18
	Micrurus surinamensis	DN76393 *		2.0e-18

	Micrurus carvalhoi	DN90798 *		2.1e-18
**Group 7**	*Micrurus lemniscatus*	DN60256 *	1F94	3.7e-17
	Micrurus surinamensis	DN65708 *		1.5e-17
	Micrurus paraensis	DN85432 *		9.3e-18
	Micrurus altirostris	AED89561 *		9.5e-18
**Subgroup 8-A**	*Micrurus corallinus*	ACS74998.1	5DO6	6.9e-16
	Micrurus spixii	DN102297/m.10825		2.9e-17
	Micrurus carvalhoi	DN62882		2.6e-18
**Subgroup 8-B**	*Micrurus corallinus*	DN122351	4RUD	1.2e-18
	Micrurus corallinus	C6JUP1		1.7e-18
	Micrurus altirostris	AED89567		6.1e-18
	Micrurus altirostris	F5CPE2 *		9.2e-18
	Micrurus altirostris	F5CPE6 *		1.5e-12
	Micrurus frontalis	P86421		1.4e-17
	Micrurus carvalhoi	DN94900		3.2e-14
**Group 9**	*Micrurus corallinus*	ACS74999	1KX1	6.3e-15
	Micrurus paraensis	DN77416		9.1e-19

Note - The HHPred lowest E-value (the best hit) structural homologue was considered as the first parameter to classify the sequences into groups. Afterwards, as for many sequences we found a rank of homologues with the same or almost the same E-value, we curated all the sequences manually considering other parameters as: primary structure homology, disulfide bond patterns, conserved structural regions (e.g. those residues close to the cysteines), and conserved functional residues. Specifically, for those sequences marked above with an asterisk (*), we used not the first HHPred option with the lowest E-value, but instead, the best option considering all the parameters analyzed manually. For all the others without the asterisk, the best HHPred option with the lowest E-value corroborates with all the manual analysis as being the best homologue.

Scheme S3. Survey of some members of the 3FT family already characterized. Information available in the literature about some 3FTs. The name of the corresponding 3FT is shown, together with the species from which the proteins were purified and/or identified in the venom, target, known activity, and the reference where this information was found. Colors are related with the type of targets.

Specie	Protein	Main Targets	Activity	References
Dendroaspis angusticeps	Tx7335	Potassium channel KcsA	Agonist	[7]
Naja kaouthia	Nk-3FTx	Potassium channel (insulinotropic activity)	Antagonist	[8]
Naja kaouthia	Cardiotoxin I	Potassium channel	Antagonist	[9,10]
Naja atra	Cardiotoxin V Cyto-toxins A1 to A6	Phospholipids of cell membrane	Cytotoxicity	[11,12]
Naja mossambica	Cytotoxins M1, M3	Phospholipids of cell membrane	Cytotoxicity	[12,13]
Naja oxiana	Cytotoxin I and II	Phospholipids of cell membrane	Cytotoxicity	[12,14]
Naja pallida	Cytotoxin Tγ	Phospholipids of cell membrane	Cytotoxicity	[12]
Bungarus candidus	Bucain	Muscarinic AChR	Hypothetical Antagonist	[15]
Bungarus multicinctus	γ-Bungarotoxin	Muscarinic AChR (M2)	Antagonist	[16, 17]
Dendroaspis augusceps	MT1 and MT2	Muscarinic AChR (M1 and M4)	Antagonist	[18]
Dendroaspis polylepis	MTα	Muscarinic AChR (M1, M2, M3, M4 and M5)	Antagonist	[18]
Naja kaouthia	WTX	Muscarinic AChR (M1, M2 and M3)	Antagonist	[19, 20]
Bungarus candidus	Candoxin	Muscle (αβγδ) and α7 neuronal type nAChRs	Antagonist	[21,22]
Bungarus multicinctus	α-bungarotoxin	Neuronal type α7, Torpedo and Muscle type (αβγδ) nAChRs	Antagonist	[21, 23, 24]
	γ-bungarotoxin	nAChR (Torpedo california)	Antagonist	[16,17]
	κ-bungarotoxin	Neuronal type nAChR (α3β2)	Antagonist	[13, 25]
Dendroaspis polylepis polylepis	α-Elapitoxin-Dpp2d	Neuronal type α7 and muscle type nAChRs	Antagonist	[26]
Laticauda semifasciata	Erabutoxin-a	Neuronal type α7, torpedo and (αβγδ) nAChRs	Antagonist	[21,22,27]
	Erabutoxin-b	nAChR	Antagonist	[28]
Ophiophagus hannah	Haditoxin	Muscle (αβγδ) and neuronal type nAChRs	Antagonist	[29]
	Oh9-1	Muscle type (rα1β1εδ) and neuronal type (rα3β2) nAChRs	Antagonist	[30]
Micrurus fulvius fulvius	Fulditoxin	Muscle type and neuronal α4β2, α7, and α3β2 nAChRs	Antagonist	[31]
Naja atra	Atratoxin and Atra-toxin-b	nAChR	Antagonist	[32]
Naja kaouthia	α-cobratoxin	Neuronal type α7, torpedo and muscle type (αβγδ) nAChRs	Antagonist	[21, 33]
	WTX	Neuronal type α7 nAChR	Antagonist	[19]
Pseudechis rossignolii	Pr-SNTX	Muscle type nAChR (α2βδε)	Antagonist	[34]
Bungarus multicinctus	α-bungarotoxin	$GABA_AR$ (α1βγ2)	Antagonist	[35]
Micrurus mipartitus	MmTx1 and MmTx2	$GABA_AR$	Positive allosteric modulator	[36]
Naja Kaouthia	α-cobratoxin	$GABA_AR$ (α1β3γ2, α1β2γ2, α2β2γ2, α5β2γ2, α2β3γ2 and α1β3δ)	Antagonist	[37]
Dendroaspis polylepis polylepis	Mambalgin-1 and 2	Acid-sensing ion channels (ASICs)	Antagonist	[38, 39]
Bungarus multicinctus	γ-Bungarotoxin	Platelet	Antagonist	[16]
Dendroaspis jamesoni kaimosae	Dendroaspin	Integrin	Antagonist	[40]
Hemachatus haemachatus	Ringhalexin	Extrinsic Tenase Complex	Inhibitor	[41]
	Hemextin	Factor VIIa	Inhibitor	[42]
	Exactin	Extrinsic Tenase Complex	Inhibitor	[43]

Naja kaouthia	KT 6.9	Platelet	Antagonist	[44]
Walterinnesia aegyptia	Actiflagelin	Spermatozoid	Interferes with motility of sperm flagellar structures	[45]

References

1. Corrêa-Netto, C.; Junqueira-de-Azevedo, I. de L.M.; Silva, D.A.; Ho, P.L.; Leitão-de-Araújo, M.; Alves, M.L.M.; Sanz, L.; Foguel, D.; Zingali, R.B.; Calvete, J.J. Snake venomics and venom gland transcriptomic analysis of Brazilian coral snakes, Micrurus altirostris and M. corallinus. J. Proteomics 2011, 74, 1795–1809, doi:10.1016/j.jprot.2011.04.003.
2. Aird, S.D.; da Silva, N.J.; Qiu, L.; Villar-Briones, A.; Saddi, V.A.; Telles, M.P. de C.; Grau, M.L.; Mikheyev, A.S. Coralsnake venomics: Analyses of venom gland transcriptomes and proteomes of six Brazilian taxa. Toxins (Basel). 2017, 9, 1–64, doi:10.3390/toxins9060187.
3. Francis, B.R.; Jorge Da Silva, N.; Seebart, C.; Casais E Silva, L.L.; Schmidt, J.J.; Kaiser, I.I. Toxins isolated from the venom of the Brazilian coral snake (Micrurus frontalis frontalis) include hemorrhagic type phospholipases A2 and postsynaptic neurotoxins. Toxicon 1997, 35, 1193–1203, doi:10.1016/S0041-0101(97)00031-7.
4. Moreira, K.G.; Prates, M. V.; Andrade, F.A.C.; Silva, L.P.; Beirão, P.S.L.; Kushmerick, C.; Naves, L.A.; Bloch, C. Frontoxins, three-finger toxins from Micrurus frontalis venom, decrease miniature endplate potential amplitude at frog neuromuscular junction. Toxicon 2010, 56, 55–63, doi:10.1016/j.toxicon.2010.02.030.
5. Hal, P.L.; Soares, M.B.; Yamanel, T.; Raw, I. REVERSE BIOLOGY APPLIED TO Micrurus corullinus, A SOUTH AMERICAN CORAL SNAKE. 1995, 14, 327–337.
6. Leão, L.I.; Ho, P.L.; Junqueira-de-Azevedo, I. de L.M. Transcriptomic basis for an antiserum against Micrurus corallinus (coral snake) venom. BMC Genomics 2009, 10, 1–14, doi:10.1186/1471-2164-10-112.
7. Rivera-Torres, I.O.; Jin, T.B.; Cadene, M.; Chait, B.T.; Poget, S.F. Discovery and characterisation of a novel toxin from Dendroaspis angusticeps, named Tx7335, that activates the potassium channel KcsA. Sci. Rep. 2016, 6, 1–11, doi:10.1038/srep23904.
8. Das, D.; Sharma, M.; Kumar Das, H.; Pratim Sahu, P.; Doley, R. Purification and Characterization of Nk-3FTx: A Three Finger Toxin from the Venom of North East Indian Monocled Cobra. J. Biochem. Mol. Toxicol. 2016, 30, 59–70, doi:10.1002/jbt.21734.
9. Nguyen, T.T.N.; Folch, B.; Létourneau, M.; Truong, N.H.; Doucet, N.; Fournier, A.; Chatenet, D. Design of a truncated cardiotoxin-I analogue with potent insulinotropic activity. J. Med. Chem. 2014, 57, 2623–2633, doi:10.1021/jm401904q.
10. Nguyen, T.T.N.; Folch, B.; Létourneau, M.; Vaudry, D.; Truong, N.H.; Doucet, N.; Chatenet, D.; Fournier, A. Cardiotoxin-I: An Unexpectedly Potent Insulinotropic Agent. ChemBioChem 2012, 13, 1805–1812, doi:10.1002/cbic.201200081.
11. Sun, Y.J.; Wu, W.G.; Chiang, C.M.; Hsin, A.Y.; Hsiao, C.D. Crystal structure of cardiotoxin V from Taiwan cobra venom: pH-dependent conformational change and a novel membrane-binding motif identified in the three-finger loops of p-type cardiotoxin. Biochemistry 1997, 36, 2403–2413, doi:10.1021/bi962594h.
12. G. Konshina, A.; V. Dubovskii, P.; G. Efremov, R. Structure and Dynamics of Cardiotoxins. Curr. Protein Pept. Sci. 2012, 13, 570–584, doi:10.2174/138920312803582960.
13. Sunagar, K.; Jackson, T.N.W.; Undheim, E.A.B.; Ali, S.A.; Antunes, A.; Fry, B.G. Three-fingered RAVERs: Rapid Accumulation of Variations in Exposed Residues of snake venom toxins. Toxins (Basel). 2013, 5, 2172–2208, doi:10.3390/toxins5112172.
14. Dubovskii, P. V.; Dubinnyi, M.A.; Konshina, A.G.; Kazakova, E.D.; Sorokoumova, G.M.; Ilyasova, T.M.; Shulepko, M.A.; Chertkova, R. V.; Lyukmanova, E.N.; Dolgikh, D.A.; et al. Structural and Dynamic "portraits" of Recombinant and Native Cytotoxin i from Naja oxiana: How Close Are They? Biochemistry 2017, 56, 4468–4477, doi:10.1021/acs.biochem.7b00453.
15. Murakami, M.; Kini, R.; Arni, R. Crystal Structure of Bucain, a Three-Fingered Toxin from the Venom of the Malayan Krait (Bungarus candidus). Protein Pept. Lett. 2009, 16, 1473–1477, doi:10.2174/092986609789839304.
16. Shiu, J.H.; Chen, C.Y.; Chang, L. Sen; Chen, Y.C.; Chen, Y.C.; Lo, Y.H.; Liu, Y.C.; Chuang, W.J. Solution structure of γ-bungarotoxin: The functional significance of amino acid residues flanking the RGD motif in integrin binding. Proteins Struct. Funct. Genet. 2004, 57, 839–849, doi:10.1002/prot.20269.
17. Chang, L.S.; Chung, C.; Wu, B.N.; Yang, C.C. Characterization and gene organization of Taiwan banded krait (Bungarus multicinctus) γ-bungarotoxin. J. Protein Chem. 2002, 21, 223–229, doi:10.1023/A:1019760401692.
18. Karlsson, E.; Jolkkonen, M.; Mulugeta, E.; Onali, P.; Adem, A. Snake toxins with high selectivity for subtypes of muscarinic acetylcholine receptors. Biochimie 2000, 82, 793–806, doi:10.1016/S0300-9084(00)01176-7.
19. Lyukmanova, E.N.; Shulepko, M.A.; Shenkarev, Z.O.; Kasheverov, I.E.; Chugunov, A.O.; Kulbatskii, D.S.; Myshkin, M.Y.; Utkin, Y.N.; Efremov, R.G.; Tsetlin, V.I.; et al. Central loop of non-conventional toxin WTX from Naja kaouthia is important for interaction with nicotinic acetylcholine receptors. Toxicon 2016, 119, 274–279, doi:10.1016/j.toxicon.2016.06.012.
20. Lyukmanova, E.N.; Shenkarev, Z.O.; Shulepko, M.A.; Paramonov, A.S.; Chugunov, A.O.; Janickova, H.; Dolejsi, E.; Dolezal, V.; Utkin, Y.N.; Tsetlin, V.I.; et al. Structural insight into specificity of interactions between nonconventional three-finger weak toxin from Naja kaouthia (WTX) and muscarinic acetylcholine receptors. J. Biol. Chem. 2015, 290, 23616–23630, doi:10.1074/jbc.M115.656595.

21. Nirthanan, S.; Gwee, M.C.E. Three-Finger α-Neurotoxins and the Nicotinic Acetylcholine Receptor, Forty Years On. J. Pharmacol. Sci. 2004, 94, 1–17, doi:10.1254/jphs.94.1.
22. Nirthanan, S.; Charpantier, E.; Gopalakrishnakone, P.; Gwee, M.C.E.; Khoo, H.E.; Cheah, L.S.; Bertrand, D.; Manjunatha Kini, R. Candoxin, a novel toxin from Bungarus candidus, is a reversible antagonist of muscle (αβγδ) but a poorly reversible antagonist of neuronal α7 nicotinic acetylcholine receptors. J. Biol. Chem. 2002, 277, 17811–17820, doi:10.1074/jbc.M111152200.
23. Utkin, Y.N. Three-finger toxins, a deadly weapon of elapid venom - Milestones of discovery. Toxicon 2013, 62, 50–55, doi:10.1016/j.toxicon.2012.09.007.
24. Dellisanti, C.D.; Yao, Y.; Stroud, J.C.; Wang, Z.Z.; Chen, L. Crystal structure of the extracellular domain of nAChR α1 bound to α-bungarotoxin at 1.94 Å resolution. Nat. Neurosci. 2007, 10, 953–962, doi:10.1038/nn1942.
25. Grant, G.A.; Luetje, C.W.; Summers, R.; Xu, X.L. Differential roles for disulfide bonds in the structural integrity and biological activity of κ-bungarotoxin, a neuronal nicotinic acetylcholine receptor antagonist. Biochemistry 1998, 37, 12166–12171, doi:10.1021/bi981227y.
26. Wang, C.I.A.; Reeks, T.; Vetter, I.; Vergara, I.; Kovtun, O.; Lewis, R.J.; Alewood, P.F.; Durek, T. Isolation and structural and pharmacological characterization of α-elapitoxin-Dpp2d, an amidated three finger toxin from black mamba venom. Biochemistry 2014, 53, 3758–3766, doi:10.1021/bi5004475.
27. Corfield, P.W.; Lee, T.J.; Low, B.W. The crystal structure of erabutoxin a at 2.0-A resolution. J. Biol. Chem. 1989, 264, 9239–9242, doi:10.1016/s0021-9258(18)60520-4.
28. Low, B.W.; Preston, H.S.; Sato, A.; Rosen, L.S.; Searl, J.E.; Rudko, A.D.; Richardson, J.S. Three dimensional structure of erabutoxin b neurotoxic protein: inhibitor of acetylcholine receptor. Proc. Natl. Acad. Sci. U. S. A. 1976, 73, 2991–2994, doi:10.1073/pnas.73.9.2991.
29. Roy, A.; Zhou, X.; Chong, M.Z.; D'Hoedt, D.; Foo, C.S.; Rajagopalan, N.; Nirthanan, S.; Bertrand, D.; Sivaraman, J.; Manjunatha Kini, R. Structural and functional characterization of a novel homodimeric three-finger neurotoxin from the venom of Ophiophagus hannah (King cobra). J. Biol. Chem. 2010, 285, 8302–8315, doi:10.1074/jbc.M109.074161.
30. Hassan-Puttaswamy, V.; Adams, D.J.; Kini, R.M. A Distinct Functional Site in ω-Neurotoxins: Novel Antagonists of Nicotinic Acetylcholine Receptors from Snake Venom. ACS Chem. Biol. 2015, 10, 2805–2815, doi:10.1021/acschembio.5b00492.
31. Foo, C.S.; Jobichen, C.; Hassan-Puttaswamy, V.; Dekan, Z.; Tae, H.S.; Bertrand, D.; Adams, D.J.; Alewood, P.F.; Sivaraman, J.; Nirthanan, S.; et al. Fulditoxin, representing a new class of dimeric snake toxins, defines novel pharmacology at nicotinic ACh receptors. Br. J. Pharmacol. 2020, 177, 1822–1840, doi:10.1111/bph.14954.
32. Lou, X.; Liu, Q.; Tu, X.; Wang, J.; Teng, M.; Niu, L.; Schuller, D.J.; Huang, Q.; Hao, Q. The atomic resolution crystal structure of atratoxin determined by single wavelength anomalous diffraction phasing. J. Biol. Chem. 2004, 279, 39094–39104, doi:10.1074/jbc.M403863200.
33. Servent, D.; Antil-Delbeke, S.; Gaillard, C.; Corringer, P.J.; Changeux, J.P.; Ménez, A. Molecular characterization of the specificity of interactions of various neurotoxins on two distinct nicotinic acetylcholine receptors. Eur. J. Pharmacol. 2000, 393, 197–204, doi:10.1016/S0014-2999(00)00095-9.
34. Yamauchi, Y.; Kimoto, H.; Yang, X.; Filkin, S.; Utkin, Y.; Kubo, T.; Inagaki, H. Pr-SNTX, a short-chain three-finger toxin from Papuan pigmy mulga snake, is an antagonist of muscle-type nicotinic acetylcholine receptor (α2βδε). Biosci. Biotechnol. Biochem. 2016, 80, 158–161, doi:10.1080/09168451.2015.1065169.
35. McCann, C.M.; Bracamontes, J.; Steinbach, J.H.; Sanes, J.R. The cholinergic antagonist α-bungarotoxin also binds and blocks a subset of GABA receptors. Proc. Natl. Acad. Sci. U. S. A. 2006, 103, 5149–5154, doi:10.1073/pnas.0600847103.
36. Rosso, J.P.; Schwarz, J.R.; Diaz-Bustamante, M.; Céard, B.; Gutiérrez, J.M.; Kneussel, M.; Pongs, O.; Bosmans, F.; Bougis, P.E. MmTX1 and MmTX2 from coral snake venom potently modulate receptor activity modulate GABAa receptor activity. Proc. Natl. Acad. Sci. U. S. A. 2015, 112, E891–E900, doi:10.1073/pnas.1415488112.
37. Kudryavtsev, D.S.; Shelukhina, I. V.; Son, L. V.; Ojomoko, L.O.; Kryukova, E. V.; Lyukmanova, E.N.; Zhmak, M.N.; Dolgikh, D.A.; Ivanov, I.A.; Kasheverov, I.E.; et al. Neurotoxins from snake venoms and α-Conotoxin ImI inhibit functionally active Ionotropic γ-aminobutyric acid (GABA) receptors. J. Biol. Chem. 2015, 290, 22747–22758, doi:10.1074/jbc.M115.648824.
38. Sun, D.; Yu, Y.; Xue, X.; Pan, M.; Wen, M.; Li, S.; Qu, Q.; Li, X.; Zhang, L.; Li, X.; et al. Cryo-EM structure of the ASIC1a-mambalgin-1 complex reveals that the peptide toxin mambalgin-1 inhibits acid-sensing ion channels through an unusual allosteric effect. Cell Discov. 2018, 4, 1–11, doi:10.1038/s41421-018-0026-1.
39. Diochot, S.; Baron, A.; Salinas, M.; Douguet, D.; Scarzello, S.; Dabert-Gay, A.S.; Debayle, D.; Friend, V.; Alloui, A.; Lazdunski, M.; et al. Black mamba venom peptides target acid-sensing ion channels to abolish pain. Nature 2012, 490, 552–555, doi:10.1038/nature11494.
40. Cheng, C.H.; Chen, Y.C.; Shiu, J.H.; Chang, Y.T.; Chang, Y.S.; Huang, C.H.; Chen, C.Y.; Chuang, W.J. Dynamics and functional differences between dendroaspin and rhodostomin: Insights into protein scaffolds in integrin recognition. Protein Sci. 2012, 21, 1872–1884, doi:10.1002/pro.2169.
41. Barnwal, B.; Jobichen, C.; Girish, V.M.; Foo, C.S.; Sivaraman, J.; Kini, R.M. Ringhalexin from Hemachatus haemachatus: A novel inhibitor of extrinsic tenase complex. Sci. Rep. 2016, 6, 1–13, doi:10.1038/srep25935.

42. Banerjee, Y.; Mizuguchi, J.; Iwanaga, S.; Kini, R.M. Hemextin AB complex, a unique anticoagulant protein complex from Hemachatus haemachatus (African Ringhals cobra) venom that inhibits clot initiation and factor VIIa activity. J. Biol. Chem. 2005, 280, 42601–42611, doi:10.1074/jbc.M508987200.
43. Girish, V.M.; Kini, R.M.R. Exactin: A specific inhibitor of Factor X activation by extrinsic tenase complex from the venom of Hemachatus haemachatus. Sci. Rep. 2016, 6, 1–13, doi:10.1038/srep32036.
44. Chanda, C.; Sarkar, A.; Sistla, S.; Chakrabarty, D. Anti-platelet activity of a three-finger toxin (3FTx) from Indian monocled cobra (Naja kaouthia) venom. Biochem. Biophys. Res. Commun. 2013, 441, 550–554, doi:10.1016/j.bbrc.2013.10.125.
45. Abd El-Aziz, T.M.; Al Khoury, S.; Jaquillard, L.; Triquigneaux, M.; Martinez, G.; Bourgoin-Voillard, S.; Sève, M.; Arnoult, C.; Beroud, R.; De Waard, M. Actiflagelin, a new sperm activator isolated from Walterinnesia aegyptia venom using phenotypic screening. J. Venom. Anim. Toxins Incl. Trop. Dis. 2018, 24, 1–11, doi:10.1186/s40409-018-0140-4.
